# Evolution of nasal and olfactory infection characteristics of SARS-CoV-2 variants

**DOI:** 10.1172/JCI174439

**Published:** 2024-03-14

**Authors:** Mengfei Chen, Andrew Pekosz, Jason S. Villano, Wenjuan Shen, Ruifeng Zhou, Heather Kulaga, Zhexuan Li, Amy Smith, Asiana Gurung, Sarah E. Beck, Kenneth W. Witwer, Joseph L. Mankowski, Murugappan Ramanathan, Nicholas R. Rowan, Andrew P. Lane

**Affiliations:** 1 Department of Otolaryngology-Head and Neck Surgery, Johns Hopkins University School of Medicine, Baltimore, Maryland, USA.; 2 Department of Molecular Microbiology and Immunology, Bloomberg School of Public Health, Baltimore, Maryland, USA.; 3 Department of Molecular and Comparative Pathobiology, Johns Hopkins University School of Medicine, Baltimore, Maryland, USA.

**Keywords:** COVID-19, Neuroscience, Neurological disorders

## Abstract

SARS-CoV-2 infection of the upper airway and the subsequent immune response are early, critical factors in COVID-19 pathogenesis. By studying infection of human biopsies in vitro and in a hamster model in vivo, we demonstrated a transition in nasal tropism from olfactory to respiratory epithelium as the virus evolved. Analyzing each variant revealed that SARS-CoV-2 WA1 or Delta infect a proportion of olfactory neurons in addition to the primary target sustentacular cells. The Delta variant possessed broader cellular invasion capacity into the submucosa, while Omicron displayed enhanced nasal respiratory infection and longer retention in the sinonasal epithelium. The olfactory neuronal infection by WA1 and the subsequent olfactory bulb transport via axon were more pronounced in younger hosts. In addition, the observed viral clearance delay and phagocytic dysfunction in aged olfactory mucosa were accompanied by a decline of phagocytosis-related genes. Further, robust basal stem cell activation contributed to neuroepithelial regeneration and restored ACE2 expression postinfection. Together, our study characterized the nasal tropism of SARS-CoV-2 strains, immune clearance, and regeneration after infection. The shifting characteristics of viral infection at the airway portal provide insight into the variability of COVID-19 clinical features, particularly long COVID, and may suggest differing strategies for early local intervention.

## Abstract 2

Tropism of SARS-CoV-2 in the nasal cavity has transitioned from olfactory to respiratory epithelium as the virus evolved.

## Graphical abstract

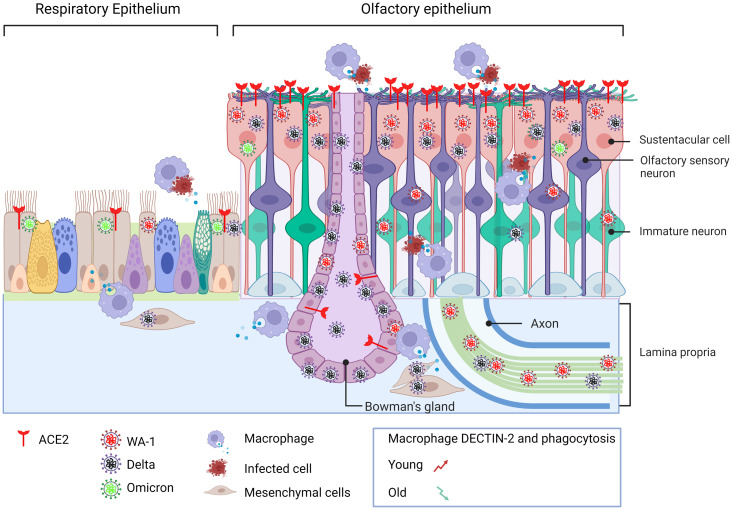

## Introduction

Severe acute respiratory syndrome coronavirus 2 (SARS-CoV-2), the causative pathogen in the worldwide pandemic of coronavirus disease 2019 (COVID-19), is readily transmitted via respiratory droplets during close contact. The nasal cavity is the entry point of the respiratory tract, and the high viral load detected there indicates that this is the principal initial site of SARS-CoV-2 infection and immune response (1, 2). The total confirmed number of Covid cases is more than 750 million globally, with a death toll reaching 7 million (3). Like other respiratory viruses, the coronavirus has mutated during its circulation around the world, with Alpha (B.1.1.7), Beta (B.1.351), Gamma (P.1), Delta (B.1.617.2), and Omicron (B.1.1.529) listed by the WHO as “Variants of Concern” (4). These variants emerged from several countries and have distinguishing traits. In the earlier part of the pandemic, the loss of the sense of smell was extremely common in patients infected with the SARS-CoV-2 original WA1 through the subsequent Delta strains. Pathological studies visualizing olfactory viral infection in postmortem samples of nasal respiratory and olfactory epithelium partially explained the smell loss in COVID-19 patients (5). In contrast, in the most recent phase of the pandemic, the Omicron variants rarely cause olfactory loss (6, 7), possibly suggesting a change in cellular tropism as the virus evolved. Systematic characterization of the SARS-CoV-2 infection pattern in the nose is important for understanding COVID-19 pathogenesis and for developing early interventions for future variants or novel respiratory viral pathogens with similar characteristics.

The cellular tropism of SARS-CoV-2 in the nasal cavity is relevant to pathologic tissue damage and to COVID testing. Cellular entry of SARS-CoV-2 depends on the binding of the virus spike S protein to angiotensin-converting enzyme 2 (ACE2) in host tissue (8, 9). The level of viral receptors and their subcellular localization is a key determinant of susceptibility to infection. In parallel with the gradually decreased ACE2 RNA expression pattern from the upper airway to distal intrapulmonary regions (10, 11), in vitro SARS-CoV-2 infection of human respiratory epithelial cell cultures shows a gradient of diminishing infectivity (olfactory epithelial cells were not included in these studies) (11). Notably, we observed up to 700-fold higher expression of ACE2 in the sustentacular cells of olfactory epithelium compared with respiratory epithelial cells in the human nose and trachea (12). SARS-CoV-2 infection in the nasal olfactory or respiratory mucosa has been visualized in postmortem samples, although quantification of the infection was not carried out because there are no validated methods to quantify scattered islands of human olfactory mucosa (5). Whether the enrichment of ACE2 in the olfactory epithelium correlates with greater susceptibility to SARS-CoV-2 infection than respiratory cells, and how the infection affects the olfactory sensory neurons and the associated olfactory bulb, need to be clarified.

Despite the substantially reduced COVID-19 incidence in adults once vaccination became widespread, cases continue to occur due to new variants, break-through infections in vaccinated or previously infected populations, and in unvaccinated populations. In 2020, SARS-CoV-2 infection caused mild acute airway illness in approximately 81% of patients with COVID-19; however, 14%–17% of hospitalized cases experienced severe symptoms and required intensive care (13). The severity of COVID-19 remains highly age-related, with a fatality rate that reached 30.5% in patients of 85 years or older early in the pandemic (14), suggesting compromised antiviral immunity with aging. The correlation between aging and the cellular damage and subsequent immune response to SARS-CoV-2 requires further investigation.

In this study, we perform in vitro infection of human nasal explants with the SARS-CoV-2 WA1 strain and show an extremely high infection rate of olfactory epithelium relative to the adjacent respiratory epithelium. By comparing the infection patterns of WA1, Delta, and Omicron strains in the hamster nasal cavity, we demonstrate a transition in tropism from olfactory to respiratory epithelium as the virus evolved, providing insight into COVID-19 pathogenesis and diagnosis. Using a WA1 strain–infected hamster model, our additional findings demonstrate an age-associated infection of olfactory neurons and impaired macrophage phagocytosis. These findings indicate that the nasal viral replication and local immune defense could be potential targets for early intervention strategies in olfactory-tropic variants.

## Results

### WA1 strain primarily targets human olfactory neuroepithelium.

The human olfactory mucosa is located in the superior part of the nasal cavity and contains olfactory sensory neurons that are responsible for the sense of smell, along with supporting sustentacular cells and neuronal progenitors. To examine the precise cellular tropism of SARS-CoV-2 in the nasal cavity including both respiratory and olfactory epithelium, we initially performed infection experiments with WA1 — the first identified strain in the US — using an ex vivo human explant culture model (15). Nasal tissue discarded in endonasal sinus and skull base surgery in individuals who were COVID-19 negative was used. To establish a reliable protocol to detect SARS-CoV-2 antigen, we screened and verified 4 different antibodies for visualizing spike (S) or nucleocapsid protein (NP) in the infected tissue sections (Supplemental Figure 1A; supplemental material available online with this article; https://doi.org/10.1172/JCI174439DS1). The staining pattern of antibodies predominantly located in apical sustentacular cells is consistent with the viral RNA detected by RNAscope analysis (Supplemental Figure 1B).

**Figure 1:**
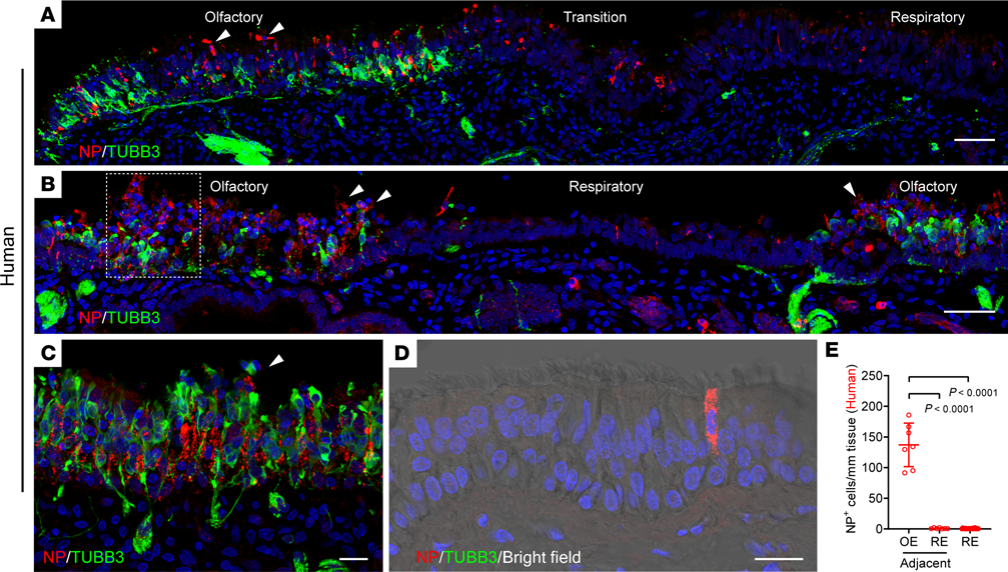
SARS-Cov-2 WA1 selectively targets human olfactory neuroepithelium. (**A** and **B**) Confocal images of SARS-CoV-2 viral antigen NP (red, Novus, NB100-56576) and olfactory neuronal marker β-III Tubulin (TUBB3, green) in superior turbinate biopsies from 2 separate patients. Images were obtained under z stack (4 μm) and tile scan mode, which covered olfactory and adjacent respiratory epithelium in the same piece of tissue. Boxed area in (**B**) was highlighted in Supplemental Figure 1, C and D. (**C** and **D**) Costaining of NP and TUBB3 in human biopsy collected from the olfactory cleft (**C**) or in a biopsy contains only respiratory epithelium (**D**). In panel **D**, NP overlapped with TUBB3^–^ ciliated cell (brightfield). Images were obtained under z stack (4 μm) and tile scan mode. (**E**) Quantification of NP^+^ cells per mm tissue. 24 independent specimens have exclusively respiratory epithelium (RE), while 7 specimens contained both respiratory and olfactory epithelium (OE). Arrowheads (**A**-**C**) indicate the detachment of infected cells. Data in **E** are represented as mean ± SD. *P* value was calculated by 1-way ANOVA. Scale bars: 50 μm (**A** and **B**); 20 μm (**C** and **D**).

Because the olfactory mucosa is irregularly distributed and surrounded by respiratory epithelium in the human nasal cavity (16), we included the immature neuronal marker TUBB3 to verify the presence of olfactory epithelium. By immunostaining with SARS-CoV-2 NP, we observed viral antigen in the TUBB3^+^ olfactory region 9 hours after infection, but very little NP in the adjacent TUBB3^–^ respiratory epithelium (Figure 1, A and B). The vast majority of NP^+^ cells colocalized with KRT18^+^ olfactory sustentacular cells (Supplemental Figure 1, C and D). Viral infection caused extensive sustentacular cell death, with rapid detachment and sloughing into the nasal lumen (Figure 1, A and B and Supplemental Figure 1, C and D). Compared with the mock control (Supplemental Figure 1E), structural damage was readily detected in the olfactory mucosa but not in the respiratory epithelium (Figure 1, A and B). A high viral infection ratio was also found in human olfactory cleft specimens obtained from skull base surgery (Figure 1C). Low viral infection was observed in 24 explants that only contained respiratory epithelium (Figure 1, D and E). We quantified the number of NP^+^ cells in 7 tissue explants, revealing 100–300-fold more infected cells in olfactory epithelium compared with adjacent respiratory epithelium (Figure 1E). Together with our earlier observed enrichment of ACE2 expression (12), this finding illustrated an olfactory-specific tropism of the SARS-CoV-2 WA1 strain, providing mechanistic insight into the pathogenesis of the pathognomonic symptom of anosmia in patients with COVID-19. Because experimental infection of human nasal explants cannot fully reflect the in vivo pathophysiology of COVID-19, we utilized a hamster infection model.

### Omicron variant shows transition in tropism from olfactory to respiratory epithelium.

As SARS-CoV-2 evolved, variants including Delta and Omicron caused surges in cases worldwide. The tropism of these different strains in the nasal cavity has not been fully clarified. Accordingly, we next characterized the cellular tropism of WA1, Delta, and Omicron in nasal mucosa using a hamster infection model. These experiments allowed us to determine whether the observed cellular tropism of WA1 in human olfactory epithelium is applicable to new variants in animal models and relates to subsequent disease pathogenesis.

**Figure 2:**
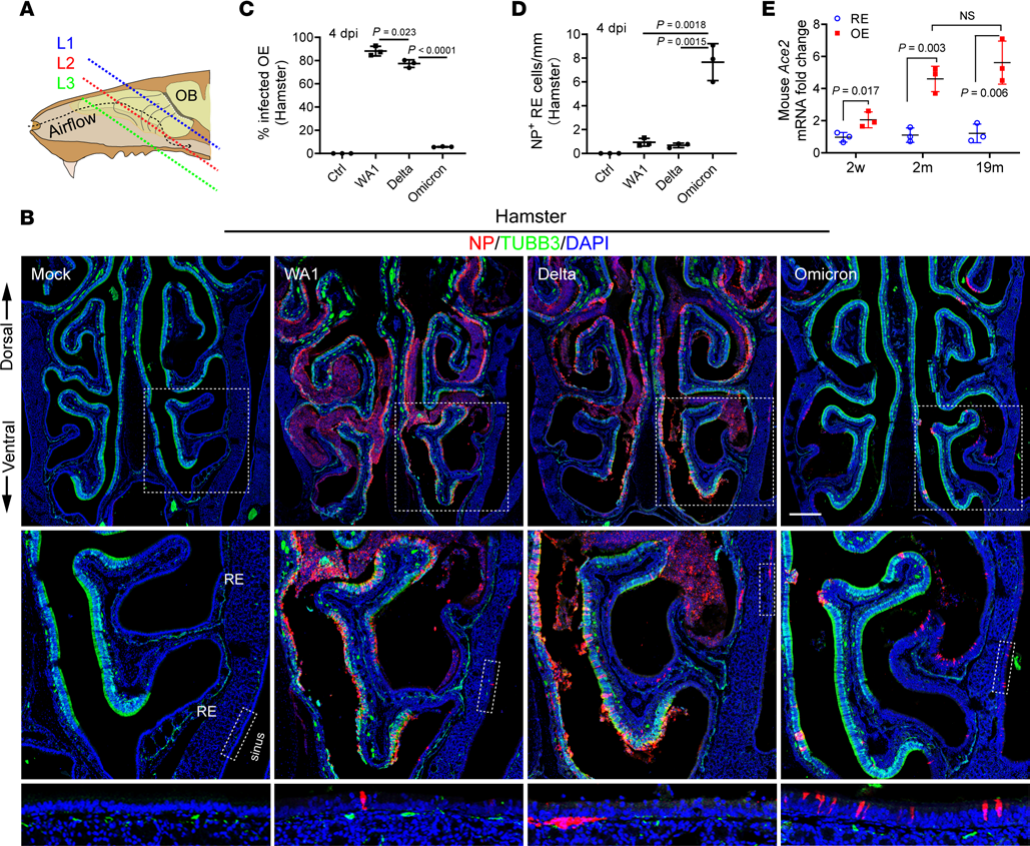
Omicron variant shows tropism transition from olfactory to respiratory epithelium. (**A**) Scheme of the tissue section. To avoid variability across different animals, frozen sections were collected and examined at 3 consistent levels (L1–3) representing the anterior (mainly respiratory epithelium), middle (respiratory + olfactory epithelium), and posterior (mainly olfactory epithelium). (**B**) Confocal images of NP and TUBB3-labeled hamster nasal sections at L2. WA1, Delta, and Omicron-infected hamsters were examined at 4 dpi. Images were obtained under z stack (12 μm) and tile scan mode. Boxed areas are highlighted at bottom. Scale bars: 500 μm. (**C**) Percentage of the infected olfactory epithelium. The total length of TUBB3^+^ or NP^+^/TUBB3^+^ epithelium in each section at L1–3 were quantified using Image J. (**D**) Quantification of NP^+^ cells in nasal respiratory epithelium. The total NP^+^ cells in TUBB3^–^ respiratory epithelium including paranasal sinuses of each section were counted. The total length of TUBB3^–^ respiratory epithelium in each section was measured using Image J. (**E**) qPCR analysis of *Ace2* expression in mouse nasal respiratory or olfactory epithelium at age of 2 weeks, 2 months, and 19 months. The entire nasal respiratory or olfactory epithelium from the same animal were isolated separately. Data are represented as mean ± S.D. Statistical significance was determined by unpaired 2-tailed *t* test. Each data point represents an individual animal (*n* = 3).

The olfactory mucosa in hamster or mouse is located in the posterior and dorsal aspect of the nasal cavity, while the anterior and ventral areas are respiratory. We confirmed that the expression of the neuronal marker TUBB3 in olfactory epithelium was mutually exclusive with the respiratory marker FOXJ1 in the nasal cavity (Supplemental Figure 2A). Therefore, the olfactory epithelium can be identified based on TUBB3^+^ staining, the presence of axon bundles, and the relatively increased thickness of the neuroepithelium. After SARS-CoV-2 inoculation (1 × 10^5^ TCID_50_ [50% tissue culture infectious dose]), we captured confocal images of the entire nasal cavity in coronal sections at 3 different levels (Figure 2A). At 4 days postinfection (dpi), we verified the extremely high viral antigen NP in the TUBB3^+^ olfactory epithelium of WA1 or Delta-infected hamsters, with an obvious decline in the adjacent respiratory epithelium (Figure 2B). About 79.2% or 70.3% of the length of TUBB3^+^ olfactory epithelium was infected by WA1 or Delta, respectively (Figure 2, B and C). We observed that the expression of ACE2 in some OMP^+^ olfactory areas was low or undetectable, perhaps correlating to the uninfected areas in WA1 or Delta treated groups (Supplemental Figure 2B).

In contrast to WA1 or Delta strains, the proportion of infected olfactory epithelium in the Omicron group was reduced to 6.7%, which is consistent with earlier reports of a comparatively lower pathogenicity in the lung of Omicron-infected hamsters (17, 18) and humans (19). The low infection rate of Omicron in olfactory epithelium (Figure 2, B and C) seems to correlate with the low incidence of smell loss in patients with Omicron infections (6, 20). Interestingly, we observed that Omicron-infected NP^+^ nasal and sinus respiratory cells were increased 7–10-fold when compared with WA1 or Delta, suggesting an olfactory-to-respiratory tropism transition with the Omicron variant (Figure 2, B and D). These tropism patterns were further demonstrated in sections of the anterior or posterior nasal cavity where the proportion of respiratory epithelium is much higher or lower, respectively (Supplemental Figure 2C and Supplemental Figure 3A). Together, these results identify that the SARS-CoV-2 variants have different tropism in nasal mucosa that may play a role in the shifting pathogenic features of COVID-19 as the virus evolved.

To support the olfactory epithelial tropism of WA1 and Delta, we further performed qPCR analysis of ACE2 expression in the entire nasal respiratory or olfactory mucosa in C57BL/6J WT mouse at the ages of 2 weeks, 2 months, and 19 months. Compared with the nasal respiratory epithelium, ACE2 mRNA transcription in adult olfactory epithelium was increased 5–7-fold in 2 month or 19 month-old animals (Figure 2E). It should be noted that ACE2 mRNA levels in whole olfactory mucosa are greatly diluted by the larger proportion of ACE2–low-to-negative cells (neurons), relative to respiratory mucosa. In addition, ACE2 protein in human (21, 22) or mouse (23) epithelial tissue is predominantly expressed at the apical surface. The more diffuse cellular pattern of ACE2 staining in human autopsy specimens may result from postmortem degradation. In any putative human olfactory tissue sample, a neuronal marker must be utilized for verification because the olfactory mucosa is irregularly distributed and surrounded by respiratory epithelium. Consistent with previous data, we observed a gradually increased ACE2 expression in olfactory mucosa from 2 weeks through adulthood (24, 25). The level of ACE2 expression in nasal respiratory mucosa was comparable between young and old animals (Figure 2E). Together, the ACE2 expression pattern supports the olfactory epithelium as a primary site of SARS-CoV-2 replication especially for WA1 or Delta variants. The decreased olfactory tropism in the Omicron variant is consistent with the recently reported endocytic entry pathway (26, 27).

### Delta variant demonstrates greater infection of cells in the nasal submucosa.

**Figure 3:**
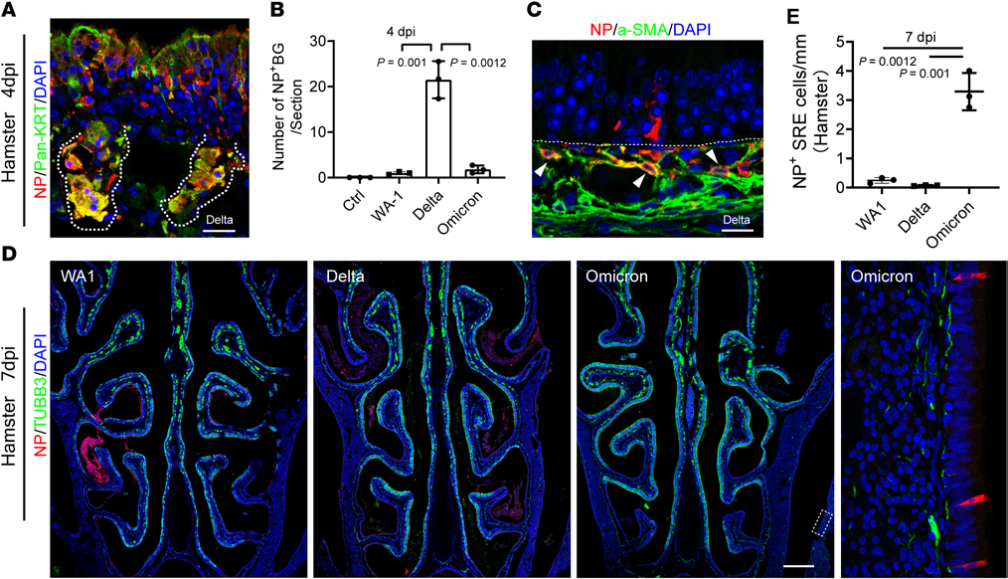
Delta variant infects cells in submucosa of the nose. (**A**) Representative image shows NP^+^/Pan-Keratin^+^ Bowman’s glands in Delta-treated hamsters. (**B**) Quantification of infected Bowman’s glands. The average number of NP^+^ Bowman’s glands in each section was calculated. 3 sections per animal were counted. (**C**) Confocal image shows NP^+^/α-SMA^+^ myofibroblasts. Hamsters infected with Delta variant at 4 dpi were examined. (**D**) Costaining of TUBB3 and NP in nasal sections at 7 dpi. Whole nasal cavity images were captured using a tile scan and z stack mode on a 14 μm section. Boxed area in Omicron-infected hamster is highlighted on the right. Scale bars: 500 μm. (**E**) Quantification of NP^+^ respiratory epithelial cells in paranasal sinuses. 3 sections per animal were counted. The total length of TUBB3^–^ respiratory epithelium in each section was measured using Image J. SRE, sinus respiratory epithelium. Images were obtained under 3 μm z stack (**A** and **C**) mode or (12 μm) z stack plus tile scan mode (**D**). Data are represented as mean ± S.D. Statistical significance was determined by unpaired 2-tailed *t* test. Each data point represents an individual animal (*n* = 3).

In the lamina propria, we frequently detected NP^+^ cells in Delta-inoculated hamsters at 4 dpi. Costaining of NP and Pancytokeratin revealed that some of those infected cells were Bowman’s glands (Figure 3A), which produce specialized mucus critical for odor perception (28). These results are in line with our previously reported ACE2 expression in human biopsies (12) and the observation that SARS-CoV-2 targets Bowman’s glands in postmortem samples by other groups (5). The number of NP^+^ Bowman’s glands in Delta-infected hamsters increased 21-fold when compared with WA1-infected hamsters, and was significantly decreased in the Omicron-infected group (Figure 3B). Additionally, NP^+^ elongated submucosa cells can be readily detected in olfactory and respiratory mucosa of Delta-infected animals (Figure 3C) but is largely reduced in Omicron-infected hamsters. These NP^+^ cells are a-SMA^+^ but negative for IBA1 (macrophage marker) and Vimentin (mesenchymal and olfactory ensheathing cell marker), suggesting the contractile myofibroblasts/mesenchymal cell lineage (Figure 3C and Supplemental Figure 3B). The broader cell types targeted in the submucosa by the Delta variant may increase the severity of tissue damage.

We next asked whether the infected submucosal cells are rapidly cleared or instead serve as an ongoing viral reservoir. At 7dpi, we observed that almost all of the NP^+^ olfactory epithelial cells had been lost, other than those in sloughed-off debris in the nasal lumen. In the submucosa, besides NP^+^ axons in the WA1 group, NP^+^ cells were barely detectable in animals infected with any of the 3 strains (Figure 3D). These results are in agreement with the reported viral titer reduction at 7dpi (17, 18). However, in the paranasal sinuses, an area that was not examined in earlier viral titer studies (17, 18), we detected a small number of NP^+^ respiratory epithelial cells in WA1 but rarely in Delta-treated hamsters at 7dpi. In parallel with the tropism transition from olfactory to respiratory epithelium, more pronounced NP^+^ sinonasal epithelial cells (3.3 positive cells/mm epithelium) were observed in Omicron variant-treated hamsters (Figure 3, D and E), suggesting a longer duration of the Omicron variant infection in sinus epithelium relative to the ancestral SARS-CoV-2 strain. Whether those Omicron-infected cells in the sinuses are actively transmitting virus at 7dpi needs to be determined.

### Age-associated SARS-CoV-2 WA1 infection of olfactory sensory neurons.

While neurological symptoms and tissue damage in olfactory cortex-related regions in MRI have been reported in patients with COVID-19 (29–31), the evidence of SARS-CoV-2 olfactory neuronal infection is controversial (5, 32). Earlier studies have shown SARS-CoV-2 RNA or viral antigen in postmortem brain tissue samples (33, 34), and rare infection observed in olfactory neurons in autopsy tissue hints toward transmucosal invasion (35). The reported data have indicated that SARS-CoV-2 infection affects olfactory sensory neurons in the hamster model (36, 37); however, TUBB3^+^-immature neurons are normally located next to the basal layer, and the long foot-like processes of infected sustentacular cells surrounding olfactory neurons could be misinterpreted in these earlier reports. The direct evidence of olfactory neuronal infection and the factors that affect the frequency of infection and entry to the brain remain to be clarified (5, 32).

**Figure 4:**
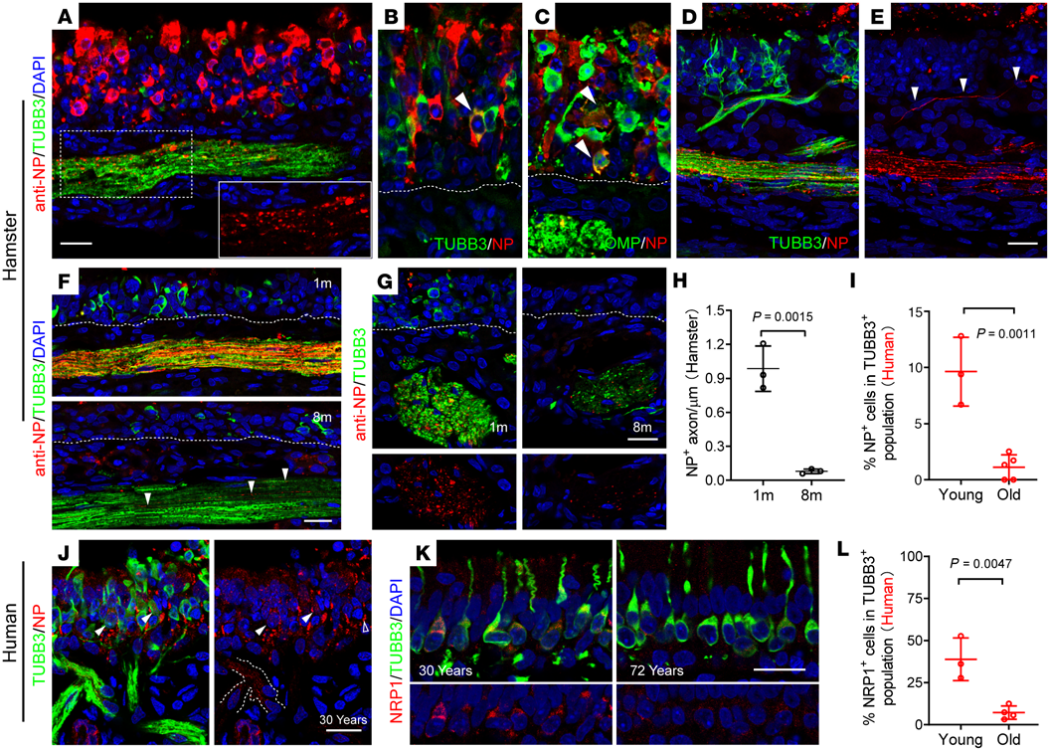
Age-associated SARS-Cov-2 infection in olfactory sensory neurons. (**A**–**C**) Confocal images showing WA1-infected hamster olfactory epithelium at 4 dpi. Insert in **A** highlighting a NP-stained axon bundle (horizontal section). Arrowheads indicate virus-infected TUBB3^+^ immature (**B**) or OMP^+^ mature (**C**) sensory neurons (coronal sections). White line indicated the basal layer of epithelium. (**D** and **E**) NP^+^ axon travel from neuroepithelium to laminar propria and merge into TUBB3^+^ axon bundle. (**F**–**H**), Quantification of NP^+^ axons in young and old hamsters at 6 dpi. Representative images show horizontal (**F**) or coronal sections (**G**). NP^+^ axons were quantified per μm of the diameter of axon bundle. (**I** and **J**) Representative images showing NP located in TUBB3^+^ human olfactory neurons (**J**) and the percentage of NP^+^ cells in TUBB3^+^ population (**I**). Dotted line in **J** indicates virus infected NP^+^ axon. Arrowheads denote NP^+^/TUBB3^+^ neurons compared with uninfected cells (empty arrowhead). Infected biopsies from 3 young donors (age 25–33 years) and 5 biopsies from older donors (age 54–72 years) were quantified for TUBB3^+^ neuronal infection. (**K** and **L**) Representative images of NRP1 expression in human olfactory epithelium (**K**) and quantification of NRP1^+^ cells in TUBB3^+^ population (**L**). 3 biopsies from young (age 20–30 years) and 4 biopsies from older donors (age 68–79 years) were examined for NRP1 expression. Images in **A**–**G** were captured with 2 μm Z-stack and exported by maximum intensity projections. Each data point represents an individual sample from hamster (**H**, *n* = 3), or human (**I** and **L**, *n* = 3-5)**.** Details of human biopsies can be found in Supplemental Table 1. Data are represented as mean ± SD. Statistical significance was determined by unpaired 2-tailed t test. Scale bars: 20 μm.

Given the high tropism of SARS-CoV-2 WA1 or Delta in olfactory mucosa, we took advantage of a hamster model to examine WA1 or Delta infection in the olfactory neuronal population. The hamster model allowed us to avoid the significant limitations of autopsy tissue, including an often prolonged and severe disease course and tissue degradation during the postmortem interval. We utilized a higher viral inoculum (1×10^7^ TCID_50_) to generate more uniform infections that would allow us to identify variation across age groups (38). As expected, we observed the vast majority of NP^+^ cells were apical sustentacular cells (39) in WA1-infected hamsters (Supplemental Figure 1, A and B) at 4 dpi. Interestingly, in the superior turbinate of the posterior nasal cavity, we observed NP labeling of a small portion of cells located in the olfactory sensory neuronal layer and their axon bundles (Figure 4A). Costaining of NP with neuronal markers TUBB3 (immature) and OMP (mature) revealed viral infection in a subset of cells from the neuronal lineage in the basal half of epithelium (Figure 4, B and C). NP^+^/OMP^+^–infected olfactory neurons were also detected in Delta variant–treated hamsters (Supplemental Figure 4A). These infected olfactory neurons were more frequently located in the superior turbinate of the posterior nasal cavity.

We detected viral antigen traveling along TUBB3^+^ axons from the epithelium to the lamina propria (Figure 4, D and E). In axon bundles, NP colocalized with TUBB3^+^ or OMP^+^ axons (Supplemental Figure 4, B and C) but did not colocalize with Vimentin^+^ ensheathing cells (Supplemental Figure 4D). In addition, we confirmed the olfactory neuronal infection by WA1 or Delta at 1 × 10^5^ TCID_50_ (Supplemental Figure 4, E and F). Precise quantification of the number of infected olfactory neurons is a challenge because SARS-CoV-2 infection elicited decay of host mRNAs and inhibition of host protein translation (5), and the epithelium had sloughed off in some areas. Compared with normal cells (Supplemental Figure 4G), the intensity of marker staining in infected and dying cells subsides. We observed at least 20 NP^+^/OMP^+^ or TUBB3^+^ olfactory neurons in each section of hamster infected with the WA1 at 1 × 10^5^ TCID_50_. Compared with WA1, olfactory neuronal infection is largely decreased in Delta and rare in the Omicron group. These data suggested that WA1 or Delta can also infect a proportion of olfactory sensory neurons, in addition to sustentacular cells that are the primary target in the upper airway. We therefore used the WA1 strain for the following aging-related experiments.

The rare expression of ACE2 protein in olfactory sensory neurons (12, 23) — which does not indicate complete absence of expression — suggests that neuronal entry may mediated by other receptors, such as Neuropilin-1(NRP1) (40, 41). In the olfactory epithelium, NRP1 was expressed in the olfactory nerve in the embryonic stage and in immature neurons after birth (42, 43). By using qPCR analysis, we detected a 2.7-fold reduction of Nrp1 mRNA in the olfactory epithelium of 19-month-old mice compared with postnatal day 14 mice (Supplemental Figure 5A). Age-related NRP1 reduction in the olfactory epithelium was also verified by IHC. About 34.2% of TUBB3^+^ olfactory neurons expressed NRP1 in young mice but only 9.7% of TUBB3^+^ neurons in the aged group displayed a low level of NRP1(Supplemental Figure 5, B–D). A few mature olfactory neurons in young mice also expressed NRP1 (Supplemental Figure 5B). In addition, NRP1 can be detected in the axon bundles and periglomerular cells in young olfactory bulbs, but are clearly declined in aged mice (Supplemental Figure 5, B and E).

The age-related pattern of NRP1 expression indicated a potential higher efficiency of SARS-CoV-2 infection in olfactory neurons in the young population. To assess whether age could be a factor mediating neuronal infection in the olfactory epithelium, we performed SARS-CoV-2 WA1 (1 × 10^7^ TCID_50_) infection experiments using young (1-month) and aged (8-month) hamsters. At 6 dpi, viral antigen (NP) could be readily detected in the axon bundles in young hamsters, but infected axons were significantly decreased in older hamsters (Figure 4, F–H). Similar to the infected neurons, TUBB3^+^ or OMP^+^ axons were more frequent in the superior turbinate of the posterior nasal cavity where Nrp1 is relatively highly expressed (43). We also examined the WA1-infected human explants and identified an increase in viral load in TUBB3^+^ neurons and axon bundles in tissue from young individuals (under 30 years old) (Figure 4, I and J). As expected, we observed 38.9% of TUBB3^+^ olfactory neurons coexpress NRP1 in younger human biopsies, but the proportion of TUBB3^+^/NRP1^+^ neurons was significantly reduced (7.2%) in older adults (Figure 4, K and L). Together, these results support age-dependent olfactory neuron infection and axonal transport.

### Increased olfactory bulb axonal transport of WA1 in young hamsters.

**Figure 5:**
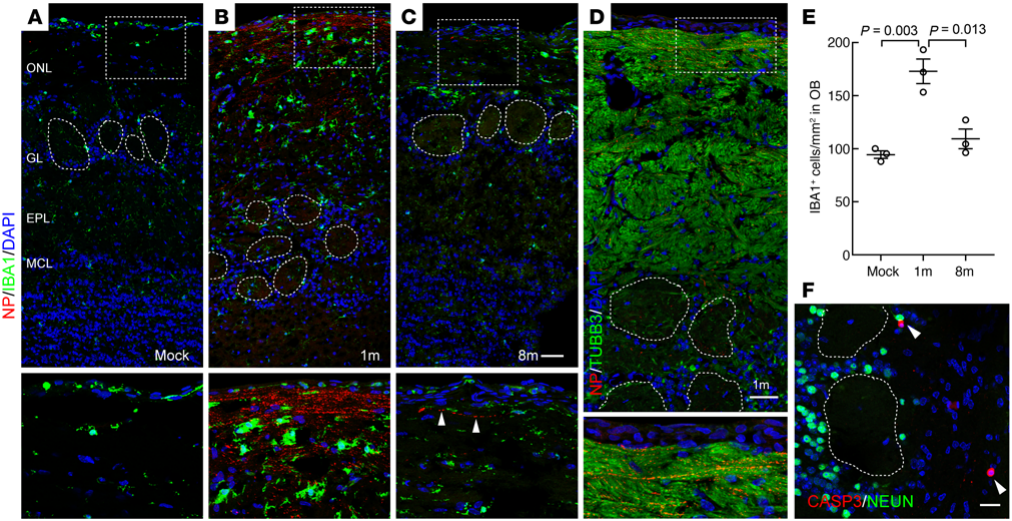
Increased olfactory bulb transport of SARS-CoV-2 in young hamsters. (**A**–**C**) Confocal images of IBA1 and NP costaining in hamster olfactory bulbs. Arrowheads indicate infected axon. (**D**) Costaining of NP and TUBB3 in a serial section next to panel **B**. (**E**) Quantification of IBA1^+^microglials in hamster olfactory bulb. Each data point in **E** (*n* = 3) represents an individual hamster sample. Data are represented as mean ± SD. Statistical significance was determined by unpaired 2-tailed *t* test. (**F**) Confocal image of cleaved caspase-3^+^/NEUN^–^ apoptotic cells (arrowheads) in the glomerular layer at 4 dpi. Images were captured with 3 μm (**A**–**D**) or 4 μm (**F**) Z-stack and exported by maximum intensity projections. Olfactory bulb tissues were collected from young and old hamsters at 6 dpi (**A**–**D**) or from mock control. Scale bars: 50 μm (**A**–**D)**; 20 μm (**F**). ONL, olfactory nerve layer; GL, glomerular layer; EPL, external plexiform layer; MCL, mitral cell layer. Boxed areas are highlighted at bottom. Dotted circles indicate glomeruli.

The increased frequency of viral NP in the axons of younger animals observed in this study indicated that SARS-CoV-2 WA1 may be prone to accessing the brain in this population. To verify this hypothesis, we examined the olfactory bulbs of 1- and 8-month-old hamsters. At 6 dpi we detected NP^+^ axons mainly located in the lateral olfactory nerve layer (ONL) in young hamsters (Figure 5, A and B) where the NRP1^+^ axons projected (42), suggesting the viral retrograde transport to olfactory bulb. Compared with the young hamsters, infected axons are rarely detected in the older group (Figure 5, A–C). Costaining analysis of serial sections verified that the NP signal is located in the TUBB3^+^ ONL (Figure 5D). In the leptomeningeal layer where the viral RNA signal was detected in postmortem samples (5), the NP antibody staining was not detectable (Figure 5, A–D). In addition, the observed leptomeningeal viral RNA staining was speculated to be extracellular virions instead of intracellular viral RNA synthesized by infected cells (5). In parallel to the greater olfactory bulb viral transport, the number of IBA1^+^ microglia cells in young olfactory bulbs was increased 1.7-fold compared with the older group (Figure 5E). It should be noted that the distribution and number of microglia in the brain changes little with age (44). We also observed that the density of IBA1^+^ microglia in uninfected olfactory bulbs is comparable between 1-month and 8-month-old animals (Supplemental Figure 5, F and G). In addition, no viral antigen could be detected in the mock control.

Immunostaining of horizontal sections crossing the olfactory mucosa and forebrain region revealed a massive number of NP^+^ axons traveling from the lateral olfactory epithelium to the olfactory bulb in young, but not aged, hamsters at 6 dpi (Supplemental Figure 6, A–F). In line with the reported NRP1 expression in the lateral olfactory nerve, which contains axons from turbinate neurons (45), the infected axons in the septal nerve was rare. NP^+^ axons could also be detected in glomeruli where the olfactory sensory neuron axon terminal projections synapse with OB mitral cells (Figure 5, B and D) at 6dpi. As a consequence of olfactory viral transport, we observed Caspase-3^+^ apoptotic cells (46) (Figure 5F, and Supplemental Figure 6G) and virus RNA in the glomerular layer at 4 dpi (Supplemental Figure 6H) in the young group. These Caspase-3^+^ cells were negative for IBA1 or the neuronal marker NEUN. The retrograde-transported virus in the olfactory bulb appears to lose the capacity for replication based on the restriction of NP signal to axons in the outer ONL and glomeruli at 6dpi (Figure 5, B–D). The detected apoptosis in the olfactory bulb may relate to infection/inflammation or loss of connection/input from the olfactory epithelium. Despite the close anatomic relationship between the olfactory mucosa and the nearby OB axons, no obvious transmucosal viral antigen NP was displayed except within axons.

Similar to ACE2 expression in lung vascular endothelial cells (47), ACE2 in the mouse or hamster olfactory bulb is mainly located in blood vessels (Supplemental Figure 6, I and J). We observed CD45^+^/IBA1^–^ immune cells infiltrating into the olfactory bulb in SARS-CoV-2–infected hACE2 mice (Supplemental Figure 6, K and L), indicating passage of leukocytes across an impaired blood-brain barrier. Given the lack of lymphatic vessels in brain parenchyma, it is unlikely that viral infection of the olfactory bulb occurs via this route (48). The microglial response in the hamster brain is not as severe as in the hACE2 mouse model, and consequently the vascular damage and the potential effect of systematic inflammation on the brain are also much milder in hamsters. Together, these results support that SARS-CoV-2 WA1 can gain access to the olfactory bulb region in the brain mainly through olfactory neuronal axons with higher frequency in the younger population, while virus replication in the olfactory bulb is limited. Of note, a recent comprehensive evaluation of 154,068 individuals with COVID-19 shows increased risks of cognition, memory disorders, and sensory disorders (including smell and taste) in younger adults (49).

### Nasal inflammatory severity is correlated with the tropism of SARS-CoV-2 variants.

The tropism of SARS-CoV-2 in the olfactory epithelium indicates the capacity of the local immune system against viral infection could be involved in the pathogenesis of COVID-19. It has been reported that reduced innate antiviral defenses, including type I and type III IFN coupled with a hyperinflammatory response is the major cause of disease severity and death in patients with COVID-19 (50). Corresponding to the high viral load in the olfactory epithelium, our qPCR analysis revealed an extensive upregulation of the antiviral gene *Ifng* (type II IFN) in the nasal turbinate tissue after infection (Supplemental Figure 7A), suggesting activated local immune defense.

**Figure 6:**
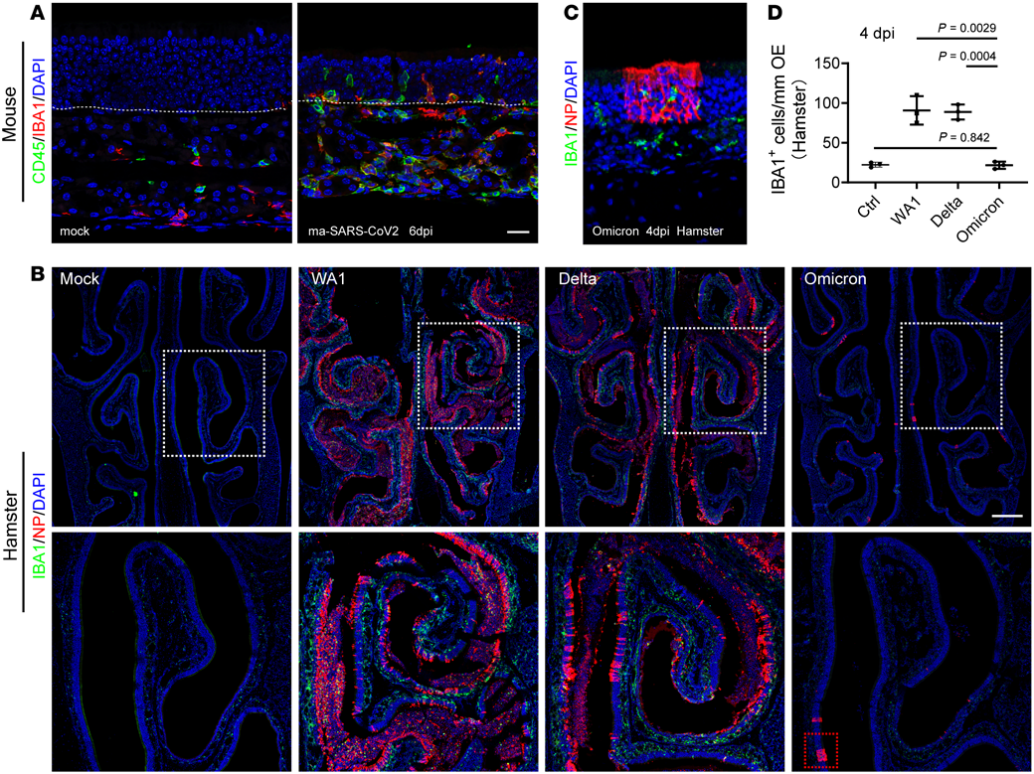
Nasal inflammatory severity is correlated with the tropism of variants. (**A**) Representative images showing CD45 and IBA1 costaining in olfactory mucosa. Mock or maSARS-Cov2 infected WT mice were examined at 6 dpi. Scale bars: 20 μm. (**B** and **C**) Confocal images of NP and IBA1-labeled hamster nasal sections. WA1, Delta, and Omicron-infected hamsters were examined at 4 dpi. Images were obtained under z stack (12 μm) and tile scan mode (**B**). White boxed areas are highlighted at bottom. Red boxed area is highlighted in **C**. Scale bars: 500 μm. (**D**) Quantification of IBA1^+^ cells in the olfactory epithelium and lamina propria. IBA1^+^ cells inside of sloughed-off debris in the nasal lumen were not included. 2 mm tissue of each section were counted. Data are represented as mean ± S.D. Statistical significance was determined by unpaired 2-tailed *t* test. Each data point represents an individual animal (*n* = 3).

Because of the limited cross-reactivity of CD45 antibodies with hamster tissue, we took advantage of the mouse-adapted SARS-CoV-2 (maSARS) infection model in C57BL/6J WT mice (51). Normally, a low number of CD45^+^ immune cells and IBA1^+^ macrophages/dendritic cells reside in the mouse olfactory mucosa. In the maSARS infected group, we observed extensive CD45^+^ immune cell infiltration into the lamina propria, crossing the basal cell layer and migrating into the neuroepithelium, suggesting a nasal immune defense in response to viral infection (Figure 6A). At 6 dpi, approximately 48.3% of CD45^+^ immune cells in olfactory mucosa were IBA1^+^ macrophages/dendritic cells, which is similar to single-cell RNA-Seq data of BALF samples from critical COVID 19 patients (52).

To compare the characteristics of the immune response in the olfactory mucosa across infection by different variants, we next quantified the number of IBA1^+^ cells in hamster model. When compared against untreated control, WA1, and Delta-stimulated IBA1^+^ cell infiltration at 4 dpi (Figure 6, B and D). In the Omicron-infected group, while coincidence of NP^+^ infected cells and IBA1^+^ cell infiltration was observed in isolated spots, the average number of IBA1^+^ cells is comparable to the untreated control (Figure 6, C and D). These results suggest that the local immune reaction to nasal cavity infection decreased as SARS-CoV-2 evolved, which is in agreement with observations of an attenuated inflammatory reaction in Omicron-infected hamster lung (17, 18).

Pneumocyte syncytia formation has been noted in patients who died from COVID-19 (53). Recent studies of SARS-CoV-2 variants highlight that spike mutations might alter viral fusogenic capacity and subsequently affect transmissibility and virus pathogenicity (54). In the WA1 or Delta-infected hamsters, we observed accumulation of NP^+^ olfactory sustentacular cells forming syncytia-like cells (Supplemental Figure 1A, Supplemental Figure 4C, and Figure 3A); however, these cells were likely dying and only present in the areas where the epithelial structure was damaged and sloughing off. In the Omicron-infected animals, these syncytia-like cells were rare due to the mild infection (Figure 6C). These results, together with previous in vitro observations (55, 56), indicated that the less efficient syncytia formation is associated with a shift in cellular tropism and altered pathogenesis in Omicron infection.

### Age-related viral clearance delay and phagocytic dysfunction in the olfactory mucosa.

**Figure 7:**
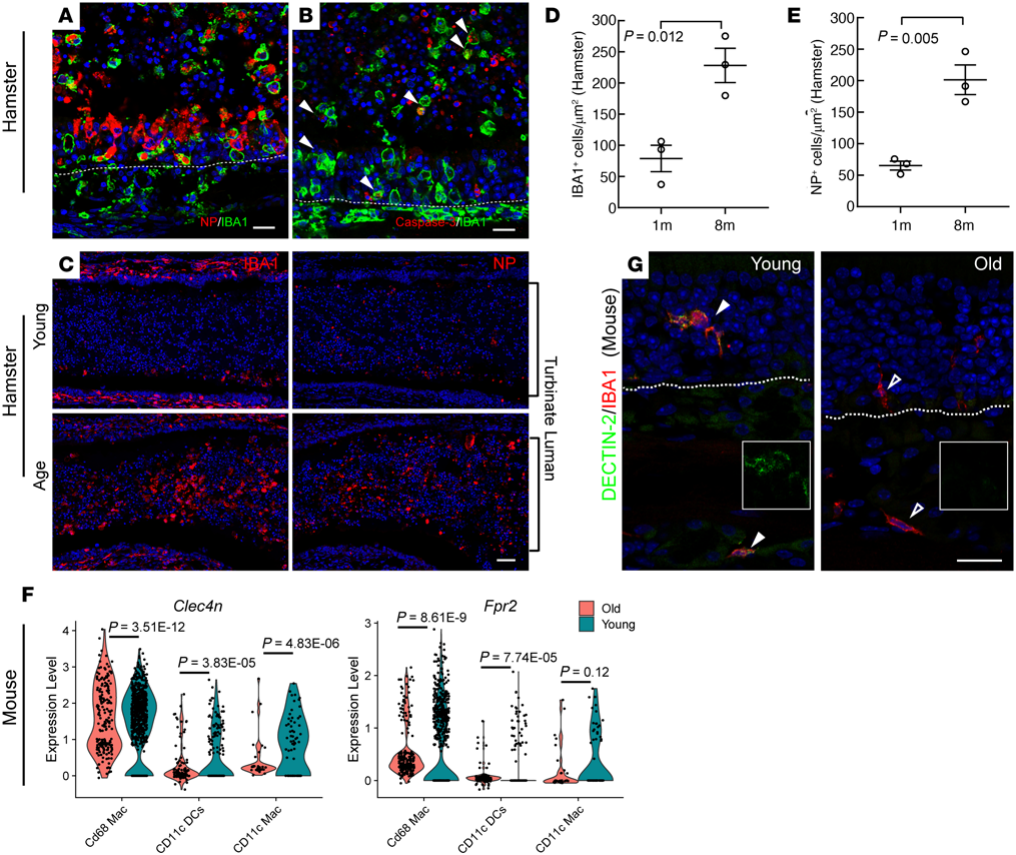
Age-associated delay in viral clearance in olfactory mucosa. (**A**) Coimmunostaining shows IBA1^+^ macrophages engulfing NP^+^ debris in hamster olfactory mucosa at 4dpi. (**B**) Representative image of IBA1 and cleaved caspase-3 costaining in hamster at 4 dpi. Arrowheads highlight the IBA1^+^ macrophages undergoing apoptosis. (**C**) Representative images showing IBA1 or NP staining in serial sections. Each panel combines 6 40× images acquired under tile scan mode. Young or old hamsters’ olfactory tissues were examined at 6dpi. (**D** and **E**) Quantification of IBA1^+^ (**D**) or NP^+^ (**E**) cells in hamster nasal olfactory lumen at 6dpi. Serial sections (**C**) from 4 different levels were quantified. (**F**) Violin plots showing the differentially expressed *Clec4n* (DECTIN-2) or *Fpr2* in young and old macrophage/dendritic lineage. (**G**) Confocal images of IBA1 and DECTIN-2 costaining in mouse olfactory mucosa. Each data point represents an individual hamster sample (**D** and **E**, *n* = 3) or an individual cell (**F**). Statistical significance was determined by an unpaired 2-tailed *t* test. The white dotted line indicates the basement membrane. Scale bars: 20 μm (**A**, **B**, and **G**); 50 μm (**C**).

We next studied the potential age-related alternation of olfactory immune response to SARS-CoV-2 infection. In hamsters, intranasal inoculation of SARS-CoV-2 induced massive shedding of NP^+^ infected cells into the nasal lumen at 4 dpi (Figure 7, A–C), consistent with our findings in infected human olfactory biopsies. IBA1^+^ macrophages/dendritic cells were widely distributed in the olfactory mucosa and the detached cells in the lumen (Figure 7, A and B). The RNA expression of *CXCL10* in hamster nasal tissue (36) or COVID-19 patients’ BALF sample (52) has been reported. Our costaining analysis showed that IBA1^+^ macrophages are the major population producing CXCL10 (Supplemental Figure 7B). Notably, 72% of IBA1^+^ cells were also positive for viral NP antigen at 4dpi (Figure 7A), indicating uptake of infected cell debris. In addition, some of the apoptotic cells sloughed into the nasal lumen were IBA1^+^/Caspase-3^+^, suggesting viral clearance by macrophages (Figure 7B).

Compared with the young hamsters, the number of IBA1^+^ cells in the nasal lumen significantly increased in the older group at 6 dpi (Figure 7, C and D). In parallel with the increased macrophages, we observed the number of remaining NP^+^ cells/debris in the serial sections of older hamster nasal cavities was increased 3.7-fold when compared with young hamsters (Figure 7, C and E), in line with the reported prolonged virus load/delayed viral clearance in older patients with COVID (57). The delayed viral clearance could be a consequence of impaired phagocytic function in aging macrophages, as reported in an influenza infection model (58).

By analyzing a previously published single cell RNA-Seq (scRNA-seq) data set (59) derived from mouse lung CD45^+^ inflammatory cells, we noted significant reduction of phagocytosis-related genes (60, 61), including *Clec4n* (DECTIN-2), *Fabp5*, *Fpr2*, and *Cd9* in old macrophage/dendritic lineages compared with young mice (Figure 7F). We further verified that the expression of DECTIN-2 was dominantly located in IBA1^+^ macrophages/dendritic cells in olfactory mucosa of young mice and declined with age (Figure 7G). Collectively, our data support that the macrophages are the critical population involved in SARS-CoV-2 defense, and their impaired viral clearance capacity could contribute to the prolonged virus retention in the olfactory mucosa of the aged population. It should be clarified that, in addition to the high proportion of IBA1^+^ macrophage/dendritic cell lineage infiltration, other inflammatory cells also contribute to antiviral immunity (62). Indeed, a recent study of olfactory epithelium biopsies revealed a diffuse infiltrate of IFN-γ expressing T cells, a shift in myeloid cell population composition, and depletion of antiinflammatory M2 macrophages in post-COVID-19 hyposmic individuals (63).

### Regenerated olfactory epithelium regains ACE2 expression.

**Figure 8:**
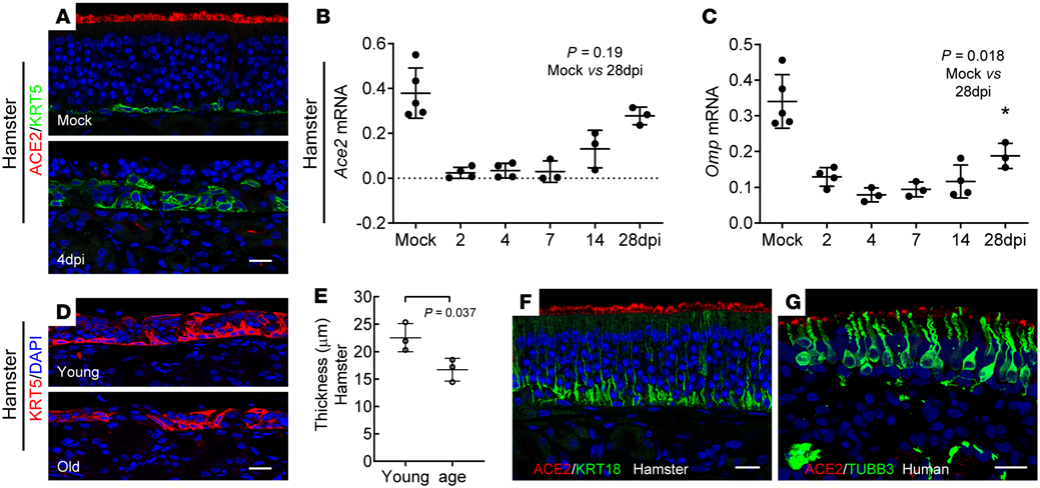
Regeneration of olfactory epithelium and reexpression of ACE2. (**A**) Confocal images showing ACE2 (red) and KRT5^+^ horizontal basal cells (green) in olfactory epithelium of mock or SARS-CoV-2 infected hamster at 4 dpi. (**B** and **C**) qPCR analysis of *Ace2* (**B**) or *Omp* (**C**) mRNA expression in SARS-CoV-2 infected hamster turbinate lysate at indicated time points. (**D** and **E**) Representative images of KRT5^+^ cells in newly regenerated olfactory epithelium (**D**) at 6 dpi, and quantification of epithelium thickness (**E**). The thickness of septal olfactory epithelium was measured using Zen lite “line” function. For each section, 8 spots were measured randomly. (**F**) Confocal image showing regenerated hamster olfactory epithelium expression of ACE2 at 28 dpi. (**G**) Representative image shows ACE2 and TUBB3^+^olfactory neurons in an olfactory biopsy from a patient with COVID-19 on day 12 after diagnosis. Dots in graph represent independent animal (**B** and **C**, *n* = 3–5; **E**, *n* = 3). Data are represented as mean ± SD. *P* value was calculated by unpaired 2-tailed Student’s *t* test. Scale bars: 20 μm.

Given the robust reparative capacity of the olfactory mucosa (64) and the rapid reconstitution after SARS-CoV2 infection (39, 65), we next systematically examined postviral stem cell–mediated regeneration using an adult hamster model (2-month old). As a consequence of viral infection, nearly complete loss of neuroepithelium was observed at 4 dpi, and ACE2 was not detectable in newly regenerated epithelium (Figure 8A). Compared with the single layer of KRT5-expressing olfactory stem cells in mock control, SARS-CoV-2 induced widespread epithelial damage and activated robust basal cell proliferation simultaneously (Supplemental Figure 7C). qPCR analysis revealed that the increased expression of *Sox2* (basal cell /sustentacular cell marker), *Lgr5* (globose basal cell marker), and *Tubb3* (immature neuron marker) was coincident with gradual reexpression of ACE2 as olfactory epithelium regeneration proceeded (Figure 8B and Supplemental Figure 7D). The expression of *Ace2* and the olfactory sensory neuron marker, *Omp*, recovered to 78% and 56% of mock at 28 dpi, respectively (Figure 8, B and C). The incomplete recovery of OMP at 28 dpi partially may explain the slow return of olfactory function in human cases with severe damage.

Coincident with epithelial repair, production of CXCL10 in IBA1^+^ macrophages vanished in both the young and old groups at 6 dpi (Supplemental Figure 7B). Compared with the old group, the newly regenerated olfactory epithelium in young hamsters was significantly thicker at 6 dpi (Figure 8, D and E), suggesting age-related delay in regeneration after infection. Further, recovery of ACE2 protein could be detected in hamsters at 28 dpi, and ACE2 expression was also observed in a patient with COVID-19 who had lost the sense of smell (Figure 8, F and G).

## Discussion

Understanding the cellular tropism and properties of SARS-CoV-2 infection of the upper airway could provide valuable insights for predicting the pathogenicity of new variants. Consistent with the enrichment of ACE2 in human olfactory sustentacular cells (12), we herein present greatly enhanced infection efficiency in human and hamster olfactory epithelium, suggesting that this site is potentially critical for initial SARS-CoV-2 infection and replication, especially for the WA1 and Delta strains. The transition of cellular tropism from olfactory to respiratory observed in the Omicron variant may help to explain the low prevalence of anosmia, while the extended duration that Omicron resides in the sinonasal respiratory epithelium may contribute to increased transmission. Our observations, together with the clinical findings of high viral loads in the nasal passages of patients with COVID-19 (1, 2), suggests that the nasal cavity is an important site of SARS-CoV-2 infection and is associated with cellular damage and host immune reaction.

The mechanisms underlying olfactory loss in SARS-CoV-2 infection are difficult to disentangle from a number of pathological processes at multiple pathophysiologic and anatomic levels (32). Quantification of SARS-CoV-2 in nasal and throat swabs reveals a gradual decrease in viral load soon after symptom onset (2, 66), suggesting a short pathologic process in the nose. Together with these findings, the rapid detachment of infected olfactory epithelium presented here may explain variation in viral loads detected on nasal swabs (2). The subsequent neuroepithelial structural damage upon viral targeting of supporting sustentacular cells and olfactory neurons plausibly underlies the high incidence of olfactory dysfunction in patients with COVID-19. Importantly, the detached olfactory epithelium likely carries a large amount of virus, and shedding of these infected cells has the potential for aerosolization, exacerbating lung infection, and facilitating transmission between individuals. A prospective longitudinal study by Menni, et al. collected data from 63,002 participants by self-reporting symptoms across Delta and Omicron periods (Between June 1, 2021, and Jan 17, 2022). The results (6) showed that loss of smell was less common in participants infected during the Omicron period than the Delta period (16.7% versus 52.7%). Recent behavior studies have also shown an attenuated olfactory dysfunction after Omicron infection using hamster model (67). Together with those observations, our results supported a correlation between nasal tissue tropism and the behavior of smell impairment (68). Other factors, including the disrupted nuclear architecture, downregulated olfactory receptor expression (69), and persistent T cell-mediated inflammation (63) in mild infection, as well as the infection of Bowman’s glands (5), may also account for the olfactory dysfunction. However, the contribution of the small proportion of olfactory neurons that become infected based on our observations is likely very limited.

Whether and how SARS-CoV-2 gains access to the brain has been investigated intensively and debated widely (32). Unlike the obvious infection of the brain in hACE2-expressing mice after SARS-CoV-2 inoculation (70, 71), viral antigen in hamster brain was not detectable (37, 72), while one study recovered SARS-CoV-2 from brain tissue (73). A recent study in a hamster model showed limited viral antigen located in nasal OMP^+^ olfactory axons (36). The presence of SARS-CoV-2 RNA or viral antigen in human postmortem brain tissue reveals that the virus may access the brain even though neuronal infection is rare (33-35). To avoid the tissue autolysis associated with long postmortem intervals, a bedside endoscopic tissue harvest procedure was developed by Khan, et al. (5). In 85 postmortem samples analyzed from COVID-19 cases, even though a uniform sustentacular cell infection was visualized in the olfactory mucosa of a patient within 4 days of diagnosis, no infection in olfactory sensory neurons was identified. It should be noted that the samples in the study by Khan, et al. were limited to relatively aged (over 62 years) patients. A recent study of COVID-19 autopsies (between 26 April 2020 and 2 March 2021) observed SARS-CoV-2 RNA or protein from diverse tissues outside of the respiratory tract, including brain. This data supports widespread virus during the viremic phase. Meanwhile, the study also reported detection of SARS-CoV-2 subgenomic RNA in the brain of one juvenile individual who died without systemic inflammation, supporting another entry mechanism (74). Our study using a hamster infection model identifies the tropism of SARS-CoV-2 WA1 and Delta in olfactory epithelium and the transport of virus to the brain through olfactory neuron axons, especially in younger hosts. It should be noted that the relatively low amount of virus transported into the olfactory bulb reported here unlikely causes significant neurologic change other than microglial activation and inflammation.

Although most children and adolescents are spared from severe COVID-19, it is reported that 22% experience neurologic involvement and 12% develop life-threatening neurologic sequelae (75). Abnormal neuroimaging manifestations, including acute disseminated encephalomyelitis-like changes, were also reported in children with COVID-19 (76). Based on infection of young and old hamsters, our observations provide strong evidence that SARS-CoV-2 WA1 targets a subset of mature and immature olfactory neurons and gains access to the brain through axon transport in an age-dependent manner. These results were supported by a comprehensive cohort study of patients with COVID-19 (49) in which a higher risk of memory and cognitive disorders, sensory disorders, and other neurologic disorders in younger adults was reported. The higher proportion of NRP1^+^ olfactory neurons in the young population may be associated with the increased neuronal infection. It should be noted that a role for other SARS-CoV-2 entry molecules besides NRP1 (77) for the invasion process cannot be excluded from our data. The unique targeting of SARS-CoV-2 (WA1 and Delta strains) to a small neuronal population may have impeded discovery to date. As previously mentioned, the absence of evidence for olfactory sensory neuron infection in postmortem samples could be attributed to the older age of the cohort studied (5). The enhanced olfactory bulb viral transport and subsequent greater level of microglial infiltration in younger hosts may call for a reassessment of neurological impairment in children. Indeed, clinical evidence indicated a recurring pattern of disease with SARS-CoV2–related abnormal CNS neuroimaging in infected children without preexisting conditions (76).

In line with previous observations of aging-related deficits of macrophage phagocytosis in influenza infection models (58), the delayed SARS-CoV-2 clearance in older hamsters’ olfactory mucosa and in patients with COVID-19 may represent a compromised phagocytic function of aged macrophages. A recent RNA-Seq study of severely affected patients with COVID-19 suggests that the decreased antigen presentation capacity of macrophages may contribute to uncontrolled viral replication and tissue damage (78). The prolonged viral retention may correlate with disease severity in aged patients with COVID-19 or with increased risk of transmission. Therefore, the local immune defense in nasal olfactory and respiratory mucosa represents a potential target for early intervention and prevention.

Robust olfactory basal cell activation efficiently regenerates sustentacular cells and restores ACE2 expression. The continued ACE2 expression in the olfactory epithelium may be important, given that anti-SARS-CoV-2 antibodies decay after approximately 6 months from the onset of symptoms, especially in individuals with mild COVID-19 disease (79). The rapid restoration of ACE2 expression in the olfactory epithelium may provide an avenue for reinfection in patients who are recovered from COVID-19. In addition, the longer duration of Omicron infection in sinonasal epithelium raises the possibility that early topical intranasal treatment may accelerate viral clearance and reduce transmission.

It should be noted that the observed viral tropism in this study only represents characteristics of infection in the nasal cavity. While our observations demonstrate a high olfactory tropism of WA1 and Delta, their infection is not limited to the olfactory epithelium, and recent RNA-Seq (80) and RNAscope or IHC (5) evidence using samples from patients with COVID-19 suggests the presence of nasal respiratory epithelial infection as well. The extent to which nasal viral load affects lower respiratory infection is not known. Our study investigated a range of postinfection days to best assess stages of neuroepithelium infection (4 dpi), axon transition (6 dpi), viral clearance (7 dpi), and regeneration (28 dpi). While our data revealed an enrichment of olfactory epithelium infection in contrast with respiratory epithelium at 4 dpi, the cellular tropism of different variants at earlier time points, e.g., 2 dpi, was not examined. Detailed time course studies would be needed to determine the kinetics of viral antigen appearance in the olfactory epithelium.

Even though the specific cellular tropism in the nasal cavity for each SARS-CoV-2 strain was identified here, it remains to be determined which group of mutations in the Spike S protein is associated with altered tropism. There are many genetic alterations between variants, and it is clear that receptor binding and entry can be very different. In addition, Omicron variants induce less syncytia formation in vitro compared with WA1 and Delta, and while we do not analyze differences in syncytia formation in vivo, the WA1 and Delta variants are most likely entering cells faster and more efficiently than the Omicron variant. Given the predominance of respiratory epithelium by area in the human nasal cavity, the enhanced respiratory infection and the extended viral retention in sinus epithelium may contribute to the increased transmissibility of Omicron and calls for a reassessment of early local intervention.

## Methods

### Sex as a biological variable.

Our study examined male and female animals, and similar findings are reported for both sexes.

### Human nasal explant in vitro infection.

Nasal biopsies, including olfactory epithelial and/or respiratory epithelial samples, were collected from patients with chronic rhinosinusitis (CRS) and people in a control group undergoing endonasal surgical approaches for non-CRS disease processes (81). All patients were tested negative for COVID-19 before surgery. In this study, the majority of biopsies were taken from superior turbinates. The human olfactory mucosa is predominantly distributed on the dorsal aspect of the nasal vault (82). The superomedial portion of superior turbinate that comprises part of the olfactory cleft contains olfactory epithelium, while the inferior and lateral side is entirely respiratory epithelium. Therefore, the coronal sections of superior turbinate samples in this study include both olfactory and respiratory epithelium, with a much smaller proportion of olfactory relative to respiratory. Notably, over 60% of the superior turbinate biopsies contained solely respiratory epithelium. Other specimens were obtained from the olfactory cleft septal mucosa. More details about the clinical specimens are listed in Supplemental Table 1.

Human biopsies were placed in PneumaCult medium (Stemcell Technologies) and sent for infection immediately. SARS-CoV-2 infection experiments were conducted in a biosafety level 3 facility at the Bloomberg School of Public Health, Johns Hopkins University. After 2 hours incubation with SARS-CoV-2/USA/WA1/2020 (BEI Resources) at 5 × 10^5^ TCID_50_/mL, the tissues were washed in PBS and transferred into fresh medium at 37°C. Mock controls were maintained in medium without virus. Tissues were fixed 9 hours after infection in 4% PFA at 4°C for 24 hours. We chose 9 hours after infection to ensure that we were within the first cycle of replication of SARS-CoV-2, and therefore, capturing the cells initially infected with the virus. The cell viability of the tissue deteriorated rapidly after approximately 12 hours irrespective of infection. Six control (2 women and 4 men ranging 45–63 years old) and 27 CRS biopsies (11 women and 16 men ranging 25–76 years old) were used for detailed IHC analysis.

Human biopsies for NRP1 staining were collected from 3 young (20–30 years) and 4 older (68–79 years) individuals. Tissues were fixed in 4% PFA at 4°C overnight, and the olfactory neuroepithelium identity was verified by TUBB3 staining.

### Animal in vivo infection.

Animal infection experiments were carried out in a biosafety level 3 facility at Johns Hopkins Research Animal Resources (RAR) in compliance with the established ethical guidelines, using WT C57BL/6J mice (Jackson Laboratory), Syrian golden hamsters (HsdHan: AURA, Envigo), and hACE2 mice (B6.Cg-Tg(KRT18-ACE2)2Prlmn/J, JAX). While both female and male animals were used in this study, animals with the same sex were used for each set of experiments. In hACE2 strain, the human ACE2 was driven by the mouse *Krt18* promoter. 6.3 × 10^5^ PFU in 20 μL (10 μL per nare) was administered intranasally to hACE2 mice. The mouse-adapted SARS-CoV-2 (courtesy of Michael Schotsaert, Icahn School of Medicine at Mt. Sinai, New York, New York, USA) infection (51) was performed as 10 μL per nare, 2.5 × 10^8^ PFU.

For hamster infection, SARS-CoV-2 in 100 μL DMEM was intranasally inoculated to hamsters (50 μL per nare). Reported viral inoculation doses ranged from 1 × 10^2^ to 1 × 10^7^ TCID_50_/mL (38, 69, 83–86) in the literature for different study purposes. However, an ideal dose should mimic the uniform infection in human olfactory epithelium, as displayed in patients with COVID (Figure 5 and 6, by Khan, et al.) (5). We used 1 × 10^5^ TCID_50_/mL of SARS-CoV-2/USA/WA1/2020, Delta variant (SARS-CoV-2/USA/MD- HP05660/2021 B.1.617.2), or Omicron (SARS-CoV-2/USA/MD-HP20874-PIDUYWZOWA/2021, BA.1.18) for infection. A higher dose of 1 × 10^7^ TCID_50_/mL of WA1 or 1 × 10^7^ TCID_50_/mL of the Delta variant was also used as indicated. The higher dose mediated a more uniform infection in the olfactory epithelium, which allowed us to compare the small number of neuronal infections among different age groups. In this study, comparisons across different variants were conducted at the same dose of infection. A detailed comparison of nasal pathology of the same variant infection with different doses was not conducted; however, we observed more uniform infection and severe damage with 1 × 10^7^ versus 1 × 10^5^ TCID_50_. Mock control animals received an equivalent volume of DMEM alone.

### Tissue processing.

Animals were euthanized in biosafety level 3 facility at indicated time points. After being anesthetized with avertin and transcardially perfused with PBS followed by 4% PFA, the skull bone was removed, and the head was postfixed in 4% PFA at 4°C for 3 days. After decalcification in TBD2 solution (Thermo Fisher Scientific) overnight and washing in PBS, tissues were equilibrated sequentially in 15% and 30% sucrose, then embedded in Optimum Cutting Temperature (OCT, Tissue-Tek) for sectioning. Fixed human biopsies were processed similarly and embedded in OCT without TBD2 treatment. Frozen sections were processed at 12 μm using MICROM HM560 cryostat (Thermo Fisher Scientific).

### IHC.

The immunostaining process was carried out on cryosections after an antigen retrieval step. Briefly, sections were washed in PBS and then blocked in 2% BSA containing 0.2% Triton X-100 at 4°C overnight, followed by incubation with primary antibodies at 4°C overnight. The primary and secondary antibodies are provided in Supplemental Methods.

### In situ hybridization.

To detect SARS-CoV-2 RNA, in situ hybridization (ISH) was performed on 12 μm–thick sections of 4% PFA-fixed OCT mounted on charged glass slides using the Leica Bond RX automated system (Leica Biosystems). Heat-induced epitope retrieval (HIER) was conducted by heating slides to 95°C for 15 minutes in EDTA-based ER2 buffer (Leica Biosystems). Slides were treated in protease (Advanced Cell Diagnostics) for 15 minutes and probes were hybridized to RNA for 1 minute. The SARS-CoV-2 probe (no. 848568, Advanced Cell Diagnostics) was detected using the Leica RNAScope 2.5 LS Assay-RED kit with a hematoxylin counterstain (no. 322150, Leica Biosystems). An RNApol2 probe served as a host gene control to evaluate RNA quality; a probe for the bacterial dap2 gene served as a negative control ISH probe.

### Confocal Imaging and Quantification.

Immunostaining images were obtained using a Zeiss LSM 780 confocal microscope equipped with a 40 ×, numerical aperture 1.1 water objective. The following laser lines were used DPSS 561 nm (detection range 560–612nm) for Alexa Fluor 546; Diode 405nm (detection range 410–480nm) for DAPI; and Argon 488nm (detection range 490–550nm) for Alexa Fluor 488. The setting of pinhole was 1 AU. Images for the same primary antibody across different samples were acquired and exported under the same settings. Before exporting, contrast adjustment was applied as necessary for individual channels using Zen lite (Zeiss) under the “Display” option. Images were cut by Photoshop and assembled by Illustrator.

For quantification, at least 5 images were collected from each specimen using 40 × objectives under the tile scan and z stack mode at same depth. Positive cells were identified according to the subcellular staining pattern and were counted manually using “Events” function of Zen lite (https://www.youtube.com/watch?v=eTPUQmYtypI). Cells in olfactory or respiratory mucosa were quantified per mm of surface epithelium by measuring the whole length of TUBB3^+^ epithelium. The SARS-CoV2 infected axons were quantified per μm diameter of axon bundle as previously described (87). Microglia in the olfactory bulb or shedding cells in the nasal cavity were quantified per mm^2^ tissue. For each patient biopsy, at least 100 TUBB3^+^ cells were quantified for calculating the percentage of NRP1^+^ cells. The quantifications were reproduced by other investigators but not blinded.

### RNA isolation, cDNA synthesis, and qPCR.

Total RNA was isolated from hamster olfactory tissue lysate using a Direct-zol RNA Kits (Zymo). Equal amounts of RNA were transcribed into cDNA by High-Capacity cDNA Reverse Transcription Kit (Applied Biosystems). On-Column DNase I digestion was conducted to remove genomic DNA contamination. Ten nanograms of cDNA were added to a 20-μL PCR reaction using SYBR Green PCR Master Mix or TaqMan Fast Universal PCR Master Mix (Applied Biosystems) on StepOne Plus System (Applied Biosystems). For SYBR Green PCR, postamplification melting curve analysis was performed to monitor unspecific products. Fold change in mRNA expression was calculated using the comparative cycle method (2^−ΔΔCt^). SYBR Green PCR primer sequences are listed in Supplemental Table 2.

### Single cell RNA-Seq analysis.

The Seurat R package was used for subsequent analysis. Quantity control was conducted according to the standard preprocessing workflow. Cells in young and old data sets express 500–2500 genes, mitochondrial genes less than 5% were selected and normalized using a scaling factor of 10,000. The highly variable genes in each data set were selected using the *FindVariableFeatures* function, and combined (10,228 cells in total) for Seurat integration procedure. The top 2,000 most variable genes per data set were used for downstream PCA and clustered using the *FindClusters* function. The data sets include 16 clusters were then projected as UMAP plots. According to the expression levels of canonical marker genes, we matched the clusters to known immune cell types. We applied *FindMarkers* function to identify differentially expressed genes between young and old conditions.

### Statistics.

Data are expressed as mean ± SD as indicated. Data analyses were carried out using GraphPad Prism. For experiments with 2 groups, *P* values were calculated using the unpaired 2-tailed Student’s *t* test. Differences were considered significant when *P* < 0.05.

### Study approval.

The research protocol involving human specimens was approved by the Johns Hopkins IRB, and all participants provided signed informed consent. Animal experimental procedures were approved by the Johns Hopkins Animal Care and Use Committee.

### Data availability.

The original images are available for public access at: https://livejohnshopkins-my.sharepoint.com/:f:/g/personal/mchen85_jh_edu/Ek_raEjP8DNCuqZ7WPUpM0UBT44K6D1vP-mN22qOIuFzvA?e=QEoF8O. Values for all data points in graphs are reported in the Supporting Data Values file. scRNA-Seq data set (GSE155006) was retrieved from published study by Mogilenko, et al. (59). This data set was generated from sorted lung CD45^+^ immune cells from 3 or 17-month-old mice. Other materials and R code will be made available upon request.

## Author contributions

MC, APL, and AP designed research. MC and WS performed experiments. MC performed the bioinformatic analysis of the single cell RNA-Seq data set. AP and RZ performed in vitro infection experiments. APL, NRR, MR, HK, AG, AS, and ZL collected biopsies or animal tissues. JSV, SEB, and JLM performed animal infection experiments; MC, WS, AP, JLM, KWW and APL analyzed data. MC, AP, and APL wrote the paper. MC and APL contributed equally to supervising the work.

## Supplementary Material

Supplemental data

Supporting data values

## Version 1 03/14/2024

In-Press Preview

## Version 2 04/15/2024

Electronic publication

## References

[1] Wang W (2020). Detection of SARS-CoV-2 in different types of clinical specimens. JAMA.

[2] Zou L (2020). SARS-CoV-2 viral load in upper respiratory specimens of infected patients. N Engl J Med.

[3] https://www.who.int/publications/m/item/weekly-epidemiological-update-on-covid-19---22-june-2023.

[4] Boehm E (2021). Novel SARS-CoV-2 variants: the pandemics within the pandemic. Clin Microbiol Infect.

[5] Khan M (2021). Visualizing in deceased COVID-19 patients how SARS-CoV-2 attacks the respiratory and olfactory mucosae but spares the olfactory bulb. Cell.

[6] Menni C (2022). Symptom prevalence, duration, and risk of hospital admission in individuals infected with SARS-CoV-2 during periods of omicron and delta variant dominance: a prospective observational study from the ZOE COVID Study. Lancet.

[7] Butowt R (2022). Why does the omicron variant largely spare olfactory function? Implications for the pathogenesis of anosmia in Coronavirus Disease 2019. J Infect Dis.

[8] Zhou P (2020). A pneumonia outbreak associated with a new coronavirus of probable bat origin. Nature.

[9] Hoffmann M (2020). SARS-CoV-2 cell entry depends on ACE2 and TMPRSS2 and is blocked by a clinically proven protease inhibitor. Cell.

[10] Sungnak W (2020). SARS-CoV-2 entry factors are highly expressed in nasal epithelial cells together with innate immune genes. Nat Med.

[11] Hou YJ (2020). SARS-CoV-2 reverse genetics reveals a variable infection gradient in the respiratory tract. Cell.

[12] Chen M (2020). Elevated ACE-2 expression in the olfactory neuroepithelium: implications for anosmia and upper respiratory SARS-CoV-2 entry and replication. Eur Respir J.

[13] Wu Z (2020). Characteristics of and important lessons from the Coronavirus Disease 2019 (COVID-19) outbreak in China: summary of a report of 72 314 cases from the Chinese Center for Disease Control and Prevention. JAMA.

[14] Wiersinga WJ (2020). Pathophysiology, transmission, diagnosis, and treatment of Coronavirus Disease 2019 (COVID-19): a review. JAMA.

[15] Hui KPY (2020). Tropism, replication competence, and innate immune responses of the coronavirus SARS-CoV-2 in human respiratory tract and conjunctiva: an analysis in ex-vivo and in-vitro cultures. Lancet Respir Med.

[16] Child KM (2018). The neuroregenerative capacity of olfactory stem cells is not limitless: implications for aging. J Neurosci.

[17] Halfmann PJ (2022). SARS-CoV-2 Omicron virus causes attenuated disease in mice and hamsters. Nature.

[18] Suzuki R (2022). Attenuated fusogenicity and pathogenicity of SARS-CoV-2 Omicron variant. Nature.

[19] Hirotsu Y (2023). Lung tropism in hospitalized patients following infection with SARS-CoV-2 variants from D614G to Omicron BA.2. Commun Med (Lond).

[20] Dehgani-Mobaraki P (2023). The Omicron variant of SARS-CoV-2 and its effect on the olfactory system. Int Forum Allergy Rhinol.

[21] Hamming I (2004). Tissue distribution of ACE2 protein, the functional receptor for SARS coronavirus. A first step in understanding SARS pathogenesis. J Pathol.

[22] Lee IT (2020). ACE2 localizes to the respiratory cilia and is not increased by ACE inhibitors or ARBs. Nat Commun.

[23] Brann DH (2020). Non-neuronal expression of SARS-CoV-2 entry genes in the olfactory system suggests mechanisms underlying COVID-19-associated anosmia. Sci Adv.

[24] Bunyavanich S (2020). Nasal gene expression of angiotensin-converting enzyme 2 in children and adults. JAMA.

[25] Bilinska K (2020). Expression of the SARS-CoV-2 Entry Proteins, ACE2 and TMPRSS2, in cells of the olfactory epithelium: identification of cell types and trends with age. ACS Chem Neurosci.

[26] Zhao H (2022). SARS-CoV-2 Omicron variant shows less efficient replication and fusion activity when compared with Delta variant in TMPRSS2-expressed cells. Emerg Microbes Infect.

[27] Hui KPY (2022). SARS-CoV-2 Omicron variant replication in human bronchus and lung ex vivo. Nature.

[28] Lee KH (1987). Isolation of an olfactory cDNA: similarity to retinol-binding protein suggests a role in olfaction. Science.

[29] Mao L (2020). Neurologic manifestations of hospitalized patients with Coronavirus Disease 2019 in Wuhan, China. JAMA Neurol.

[30] Varatharaj A (2020). Neurological and neuropsychiatric complications of COVID-19 in 153 patients: a UK-wide surveillance study. Lancet Psychiatry.

[31] Douaud G (2022). SARS-CoV-2 is associated with changes in brain structure in UK Biobank. Nature.

[32] Xydakis MS (2021). Post-viral effects of COVID-19 in the olfactory system and their implications. Lancet Neurol.

[33] Matschke J (2020). Neuropathology of patients with COVID-19 in Germany: a post-mortem case series. Lancet Neurol.

[34] Solomon IH (2020). Neuropathological features of Covid-19. N Engl J Med.

[35] Meinhardt J (2021). Olfactory transmucosal SARS-CoV-2 invasion as a port of central nervous system entry in individuals with COVID-19. Nat Neurosci.

[36] de Melo GD (2021). COVID-19-related anosmia is associated with viral persistence and inflammation in human olfactory epithelium and brain infection in hamsters. Sci Transl Med.

[37] Zhang AJ (2021). Severe acute respiratory syndrome coronavirus 2 infects and damages the mature and immature olfactory sensory neurons of hamsters. Clin Infect Dis.

[38] Jiang L (2022). A bacterial extracellular vesicle-based intranasal vaccine against SARS-CoV-2 protects against disease and elicits neutralizing antibodies to wild-type and Delta variants. J Extracell Vesicles.

[39] Bryche B (2020). Massive transient damage of the olfactory epithelium associated with infection of sustentacular cells by SARS-CoV-2 in golden Syrian hamsters. Brain Behav Immun.

[40] Cantuti-Castelvetri L (2020). Neuropilin-1 facilitates SARS-CoV-2 cell entry and infectivity. Science.

[41] Daly JL (2020). Neuropilin-1 is a host factor for SARS-CoV-2 infection. Science.

[42] Miller AM (2010). Axon fasciculation in the developing olfactory nerve. Neural Dev.

[43] Pasterkamp RJ (1998). Evidence for a role of the chemorepellent semaphorin III and its receptor neuropilin-1 in the regeneration of primary olfactory axons. J Neurosci.

[44] Deng XH (2006). Cytokine-induced activation of glial cells in the mouse brain is enhanced at an advanced age. Neuroscience.

[45] Schwarting GA (2000). Semaphorin 3A is required for guidance of olfactory axons in mice. J Neurosci.

[46] Kolb JP (2017). Programmed cell death and inflammation: winter is coming. Trends Immunol.

[47] Alon R (2021). Leukocyte trafficking to the lungs and beyond: lessons from influenza for COVID-19. Nat Rev Immunol.

[48] Louveau A (2018). CNS lymphatic drainage and neuroinflammation are regulated by meningeal lymphatic vasculature. Nat Neurosci.

[49] Xu E (2022). Long-term neurologic outcomes of COVID-19. Nat Med.

[50] Blanco-Melo D (2020). Imbalanced host response to SARS-CoV-2 drives development of COVID-19. Cell.

[52] Liao M (2020). Single-cell landscape of bronchoalveolar immune cells in patients with COVID-19. Nat Med.

[53] Braga L (2021). Drugs that inhibit TMEM16 proteins block SARS-CoV-2 spike-induced syncytia. Nature.

[54] Escalera A (2022). Mutations in SARS-CoV-2 variants of concern link to increased spike cleavage and virus transmission. Cell Host Microbe.

[55] Meng B (2022). Altered TMPRSS2 usage by SARS-CoV-2 Omicron impacts infectivity and fusogenicity. Nature.

[56] Willett BJ (2022). SARS-CoV-2 Omicron is an immune escape variant with an altered cell entry pathway. Nat Microbiol.

[57] Zheng S (2020). Viral load dynamics and disease severity in patients infected with SARS-CoV-2 in Zhejiang province, China, January-March 2020: retrospective cohort study. BMJ.

[58] Wong CK (2017). Aging impairs alveolar macrophage phagocytosis and increases influenza-induced mortality in mice. J Immunol.

[59] Mogilenko DA (2021). Comprehensive profiling of an aging immune system reveals clonal GZMK^+^ CD8^+^ T cells as conserved hallmark of inflammaging. Immunity.

[60] Jaitin DA (2019). Lipid-associated macrophages control metabolic homeostasis in a Trem2-dependent manner. Cell.

[61] Gordon S (2016). Phagocytosis: an immunobiologic process. Immunity.

[62] Witkowski M (2021). Untimely TGFβ responses in COVID-19 limit antiviral functions of NK cells. Nature.

[63] Finlay JB (2022). Persistent post-COVID-19 smell loss is associated with immune cell infiltration and altered gene expression in olfactory epithelium. Sci Transl Med.

[64] Schwob JE (2017). Stem and progenitor cells of the mammalian olfactory epithelium: taking poietic license. J Comp Neurol.

[65] Urata S (2021). Regeneration profiles of olfactory epithelium after SARS-CoV-2 infection in golden syrian hamsters. ACS Chem Neurosci.

[66] Wolfel R (2020). Virological assessment of hospitalized patients with COVID-2019. Nature.

[67] de Melo GD (2023). Neuroinvasion and anosmia are independent phenomena upon infection with SARS-CoV-2 and its variants. Nat Commun.

[68] Reyna RA (2022). Recovery of anosmia in hamsters infected with SARS-CoV-2 is correlated with repair of the olfactory epithelium. Sci Rep.

[69] Zazhytska M (2022). Non-cell-autonomous disruption of nuclear architecture as a potential cause of COVID-19-induced anosmia. Cell.

[70] Winkler ES (2020). SARS-CoV-2 infection of human ACE2-transgenic mice causes severe lung inflammation and impaired function. Nat Immunol.

[71] Golden JW (2020). Human angiotensin-converting enzyme 2 transgenic mice infected with SARS-CoV-2 develop severe and fatal respiratory disease. JCI Insight.

[72] Sia SF (2020). Pathogenesis and transmission of SARS-CoV-2 in golden hamsters. Nature.

[73] Imai M (2020). Syrian hamsters as a small animal model for SARS-CoV-2 infection and countermeasure development. Proc Natl Acad Sci U S A.

[74] Stein SR (2022). SARS-CoV-2 infection and persistence in the human body and brain at autopsy. Nature.

[75] LaRovere KL (2021). Neurologic Involvement in Children and Adolescents Hospitalized in the United States for COVID-19 or Multisystem Inflammatory Syndrome. JAMA Neurol.

[76] Lindan CE (2021). Neuroimaging manifestations in children with SARS-CoV-2 infection: a multinational, multicentre collaborative study. Lancet Child Adolesc Health.

[77] Peng R (2021). Cell entry by SARS-CoV-2. Trends Biochem Sci.

[78] Chen ST (2022). A shift in lung macrophage composition is associated with COVID-19 severity and recovery. Sci Transl Med.

[79] Ibarrondo FJ (2020). Rapid decay of anti-SARS-CoV-2 antibodies in persons with mild Covid-19. N Engl J Med.

[80] Ziegler CGK (2021). Impaired local intrinsic immunity to SARS-CoV-2 infection in severe COVID-19. Cell.

[81] Chen M (2019). Chronic inflammation directs an olfactory stem cell functional switch from neuroregeneration to immune defense. Cell Stem Cell.

[82] Leopold DA (2000). Anterior distribution of human olfactory epithelium. Laryngoscope.

[83] Rogers TF (2020). Isolation of potent SARS-CoV-2 neutralizing antibodies and protection from disease in a small animal model. Science.

[84] Dhakal S (2021). Sex differences in lung imaging and SARS-CoV-2 antibody responses in a COVID-19 Golden Syrian hamster Model. mBio.

[85] Mulka KR (2022). Progression and resolution of severe acute respiratory syndrome Coronavirus 2 (SARS-CoV-2) infection in Golden Syrian hamsters. Am J Pathol.

[86] Plunkard J (2023). SARS-CoV-2 variant pathogenesis following primary infection and reinfection in Syrian Hamsters. mBio.

[87] Park KK (2008). Promoting axon regeneration in the adult CNS by modulation of the PTEN/mTOR pathway. Science.

