# Supplementary material for: Evolution of nasal and olfactory infection characteristics of SARS-CoV-2 variants
Source: J Clin Invest. 2024 Mar 14;134(8):e174439. doi: 10.1172/JCI174439 (PMC11014658; doi:10.1172/JCI174439)
Supplement: Supplemental data [file jci-134-174439-s036.pdf]

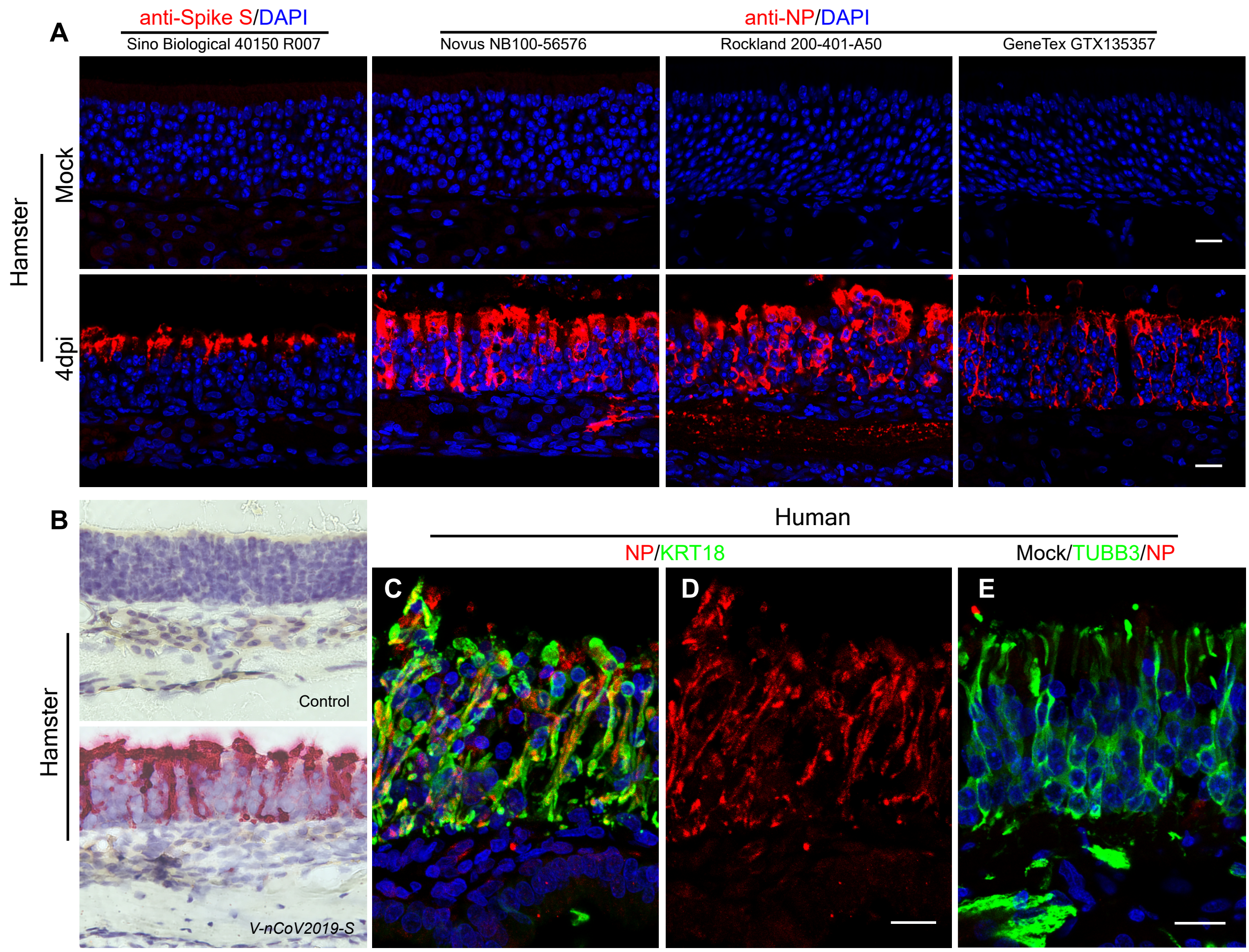


**Supplemental Figure 1. Detection of SARS-Cov-2 in the olfactory neuroepithelium.**

**(A)** SARS-Cov-2 antibody testing. 1 anti-spike S and 3 different anti-NP were verified to be reliable for frozen section immunohistochemistry. Hamster olfactory tissue was examined at 4dpi. All 4 antibodies stained in the same pattern showing intensive viral load mainly located in the apical sustentacular cell layer. No signal could be detected in mock control. Catalog number for each antibody is presented accordingly.

**(B)** RNAscope analysis showing SARS-Cov-2 viral RNA on 4dpi in hamster olfactory epithelium.

**(C** and **D)** Co-staining of NP and sustentacular cell marker KRT18. Image was captured from the boxed area in panel (**B**) of Figure 1.

**(E)** Confocal image of NP and TUBB3 staining in mock control. Scale bars: 20 μm.

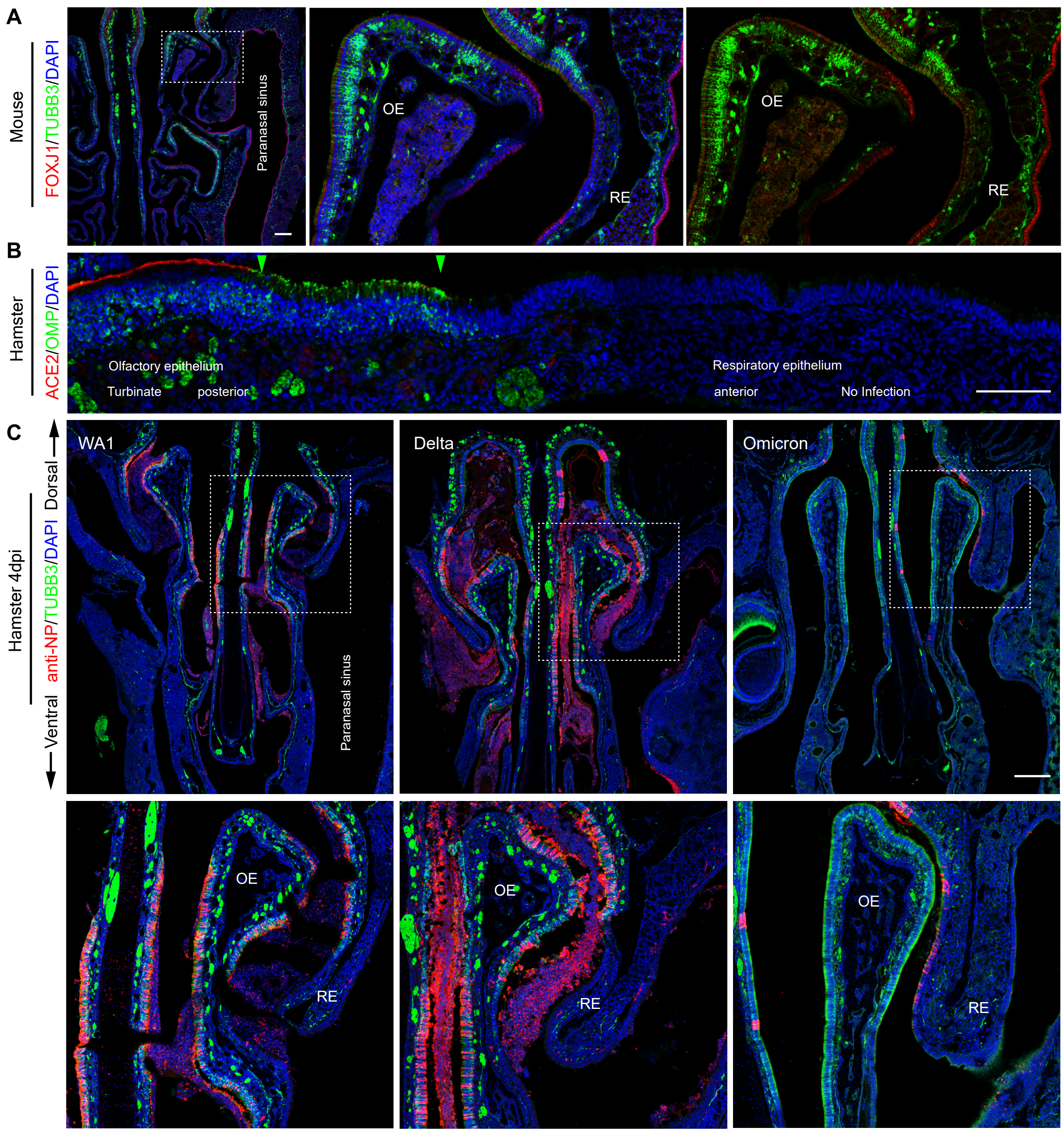


**Supplemental Figure 2. Decreased Omicron variant infection in hamster olfactory epithelium.**

(**A**) Co-staining of neuronal marker TUBB3 and respiratory epithelium marker FOXJ1 in mouse nasal cavity.
(**B**) Representative image shows OMP and rabbit anti ACE2 co-staining in hamster turbinate horizontal section. Intense ACE2 expression is seen in OMP+ olfactory epithelium. The green arrows show the respiratory-olfactory transition area with lower ACE2 expression. The intensity of ACE2 staining in respiratory is lower than olfactory epithelium and is not evenly distributed.

(**C**) Confocal images show the distribution of NP and TUBB3 in a coronal section at L1 of the nasal cavity. Tissues were examined on 4dpi, boxed areas were highlighted at bottom. Note that NP was declined from TUBB3 negative respiratory epithelium (RE) in hamsters infected with WA1 or Delta. The respiratory infection in Omicron group was markedly increased.

Images were obtainedunder 3 μm z stack (**A** and **B**) mode or (10 μm) z stack plus tile scan mode (**C**). Scale bars: 200 μm (**A**), 100 μm (**B**), and 500 μm (**C**).

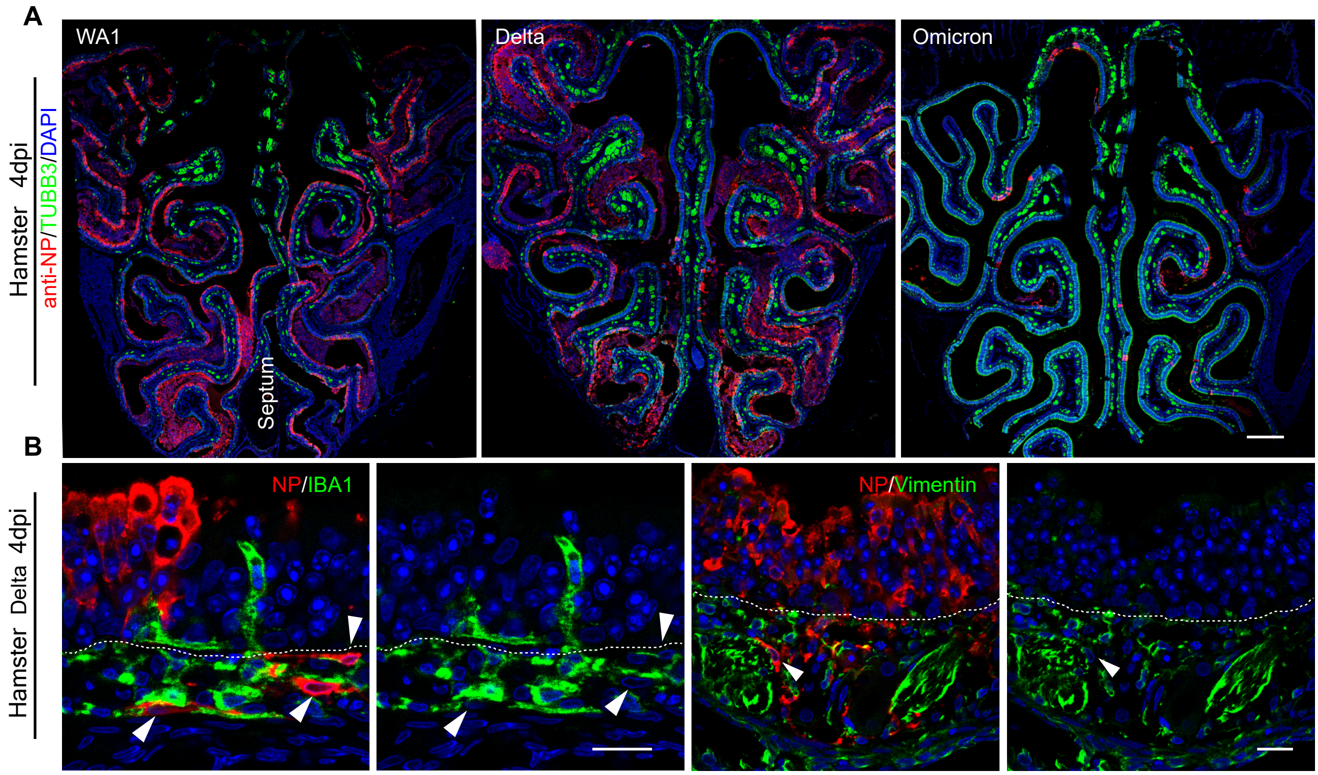


**Supplemental Figure 3. Tropism of SARS-CoV-2 variants in the posterior nasal mucosa.**

(**A**) Confocal images showing the distribution of NP and TUBB3 in posterior nasal cavity sections at 4dpi. Coronal sections at L3 were examined, where the proportion of olfactory epithelium is predominant. The olfactory epithelium infection in the Omicron group was decreased remarkably. Images were obtained under (12 μm) z stack plus tile scan mode. Scale bar = 500 μm.

(**B**) Co-staining of NP and IBA1 (macrophage marker) or NP and Vimentin (mesenchymal cell and olfactory ensheathing cell marker) in Delta infected hamster. Scale bars = 20 μm. The white dotted line in (**B**) indicates the basement membrane.

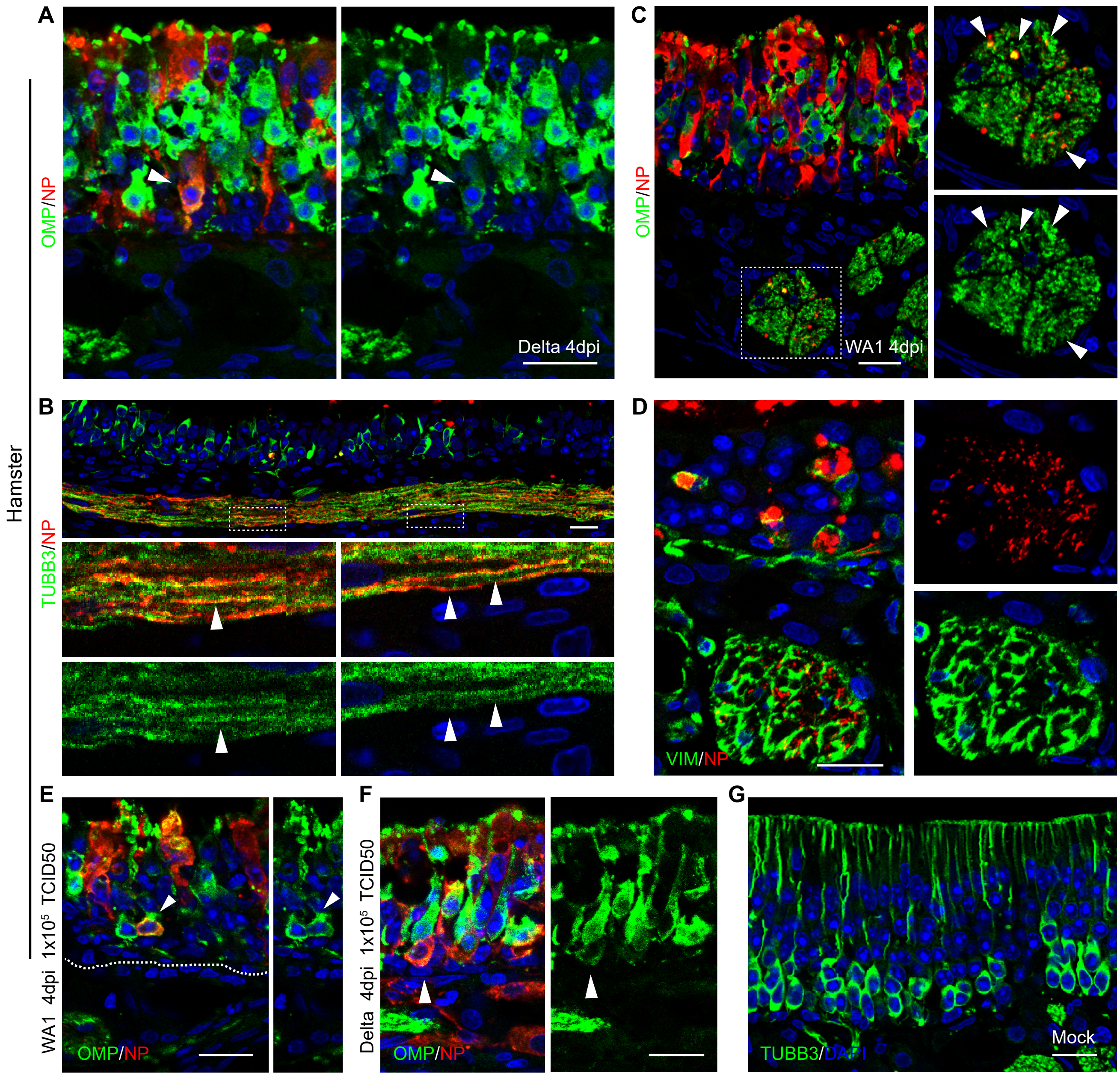


**Supplemental Figure 4. SARS-Cov-2 WA1 and Delta variants infect olfactory sensory neurons.**

(**A**) Co-staining of NP and OMP in olfactory epithelium. 5 weeks-old hamsters were infected with SARS-CoV-2-Delta variant ($1 \times 10^7$ TCID50) and were examined on 4dpi. Arrowhead indicates an infected neuron.

(**B** and **C**) Confocal images show co-localization of NP with TUBB3+ or OMP+ axon. (**B**) shows a larger view of Figure 4**F**. Boxed areas in (**B**) were highlighted at bottom. 1m (**B**) or 7-8 weeks-old (**C**) hamsters were infected with WA1.

(**D**) Representative image shows NP signal does not colocalize with Vimentin (VIM) in axon bundles.

(**E** and **F**) Co-staining of NP and OMP in olfactory epithelium.
(**G**) Confocal image shows TUBB3+ immature olfactory neurons in mock group.

7-8 week-old hamsters were infected WA1 (**D** and **E**) or Delta variant (**F**) at $1 \times 10^5$ TCID50 and were examined at 4dpi. Images (**A**-**F**) were obtained under 2 μm z stack scan mode. Scale bars: 20 μm.

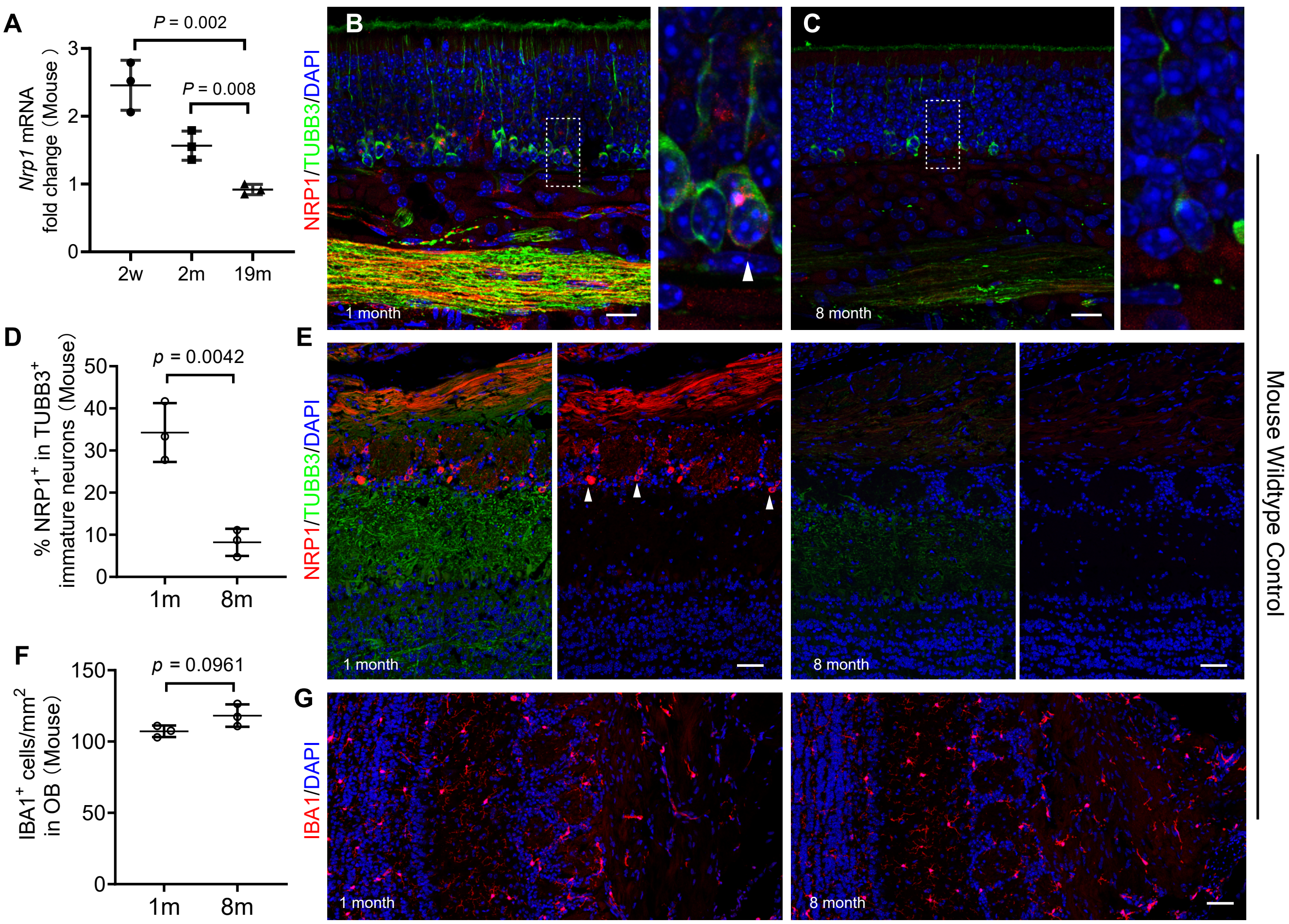


**Supplemental Figure 5. Expression of NRP1 in mouse olfactory epithelium and bulb**

(**A**) qPCR analysis of *Nrp1* expression in mouse olfactory mucosa at ages 2 weeks, 2 months, and 19 months.

(**B** and **C**) Immunostaining analysis shows the expression of NRP1 in the TUBB3+ immature olfactory neurons and axon bundles. Confocal images were acquired from horizontal section of young (1 month) and old (8 month) mice. Boxed areas were highlighted on right. In young mice, a few mature neurons above the TUBB3+ cells also express a low level of NRP1.

(**D**) Percentage of NRP1 expressing cells in TUBB3+ immature neurons. Olfactory tissues from wildtype mice were examined at the indicated age groups.

(**E**) Confocal images show the expression of NRP1 in young and old mouse olfactory bulb. Arrowheads highlight NRP1+ cells in glomerular layer.

(**F** and **G**) Quantification of IBA1+ microglial in the olfactory bulb (**F**) and representative images show the microglial distribution in the lateral olfactory bulb (**G**).

Each data point represents an individual mouse (n=3). Data are represented as mean ± S.D. Statistical significance was determined by unpaired two-tailed t-test. Scale bars: 20 µm (**B** and **C**); 50 µm (**E** and **G**) .

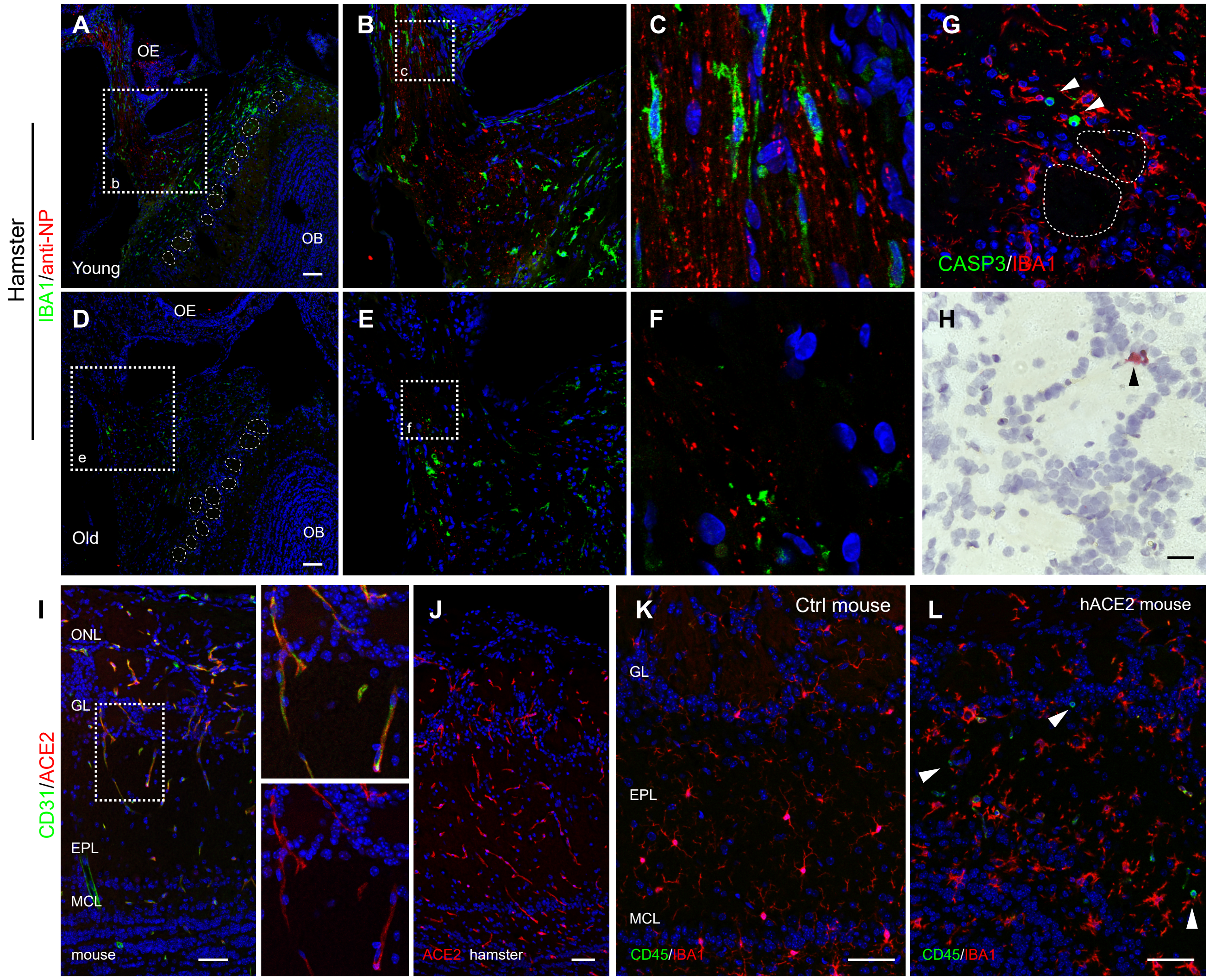


**Supplemental Figure 6. Increased brain transport of SARS-CoV-2 in young hamsters**

(**A-F**) Confocal image capturing a cross section of olfactory epithelium and olfactory bulb. SARS-CoV-2-infected young or old hamsters were examined at 6 dpi. Boxed areas highlight the infected lateral olfactory axons crossing the cribriform plate and projecting to the olfactory bulb. The NP+ axons involved in the olfactory nerve layer are not distributed evenly as a 3D axon projection in the olfactory bulb. Images were captured with 3 µm Z-stack and exported by maximum intensity projections. OE, olfactory epithelium; OB, olfactory bulb.

(**G**) Co-staining of Caspase-3 and IBA1 in olfactory bulb at 4dpi.
(**H**) RNAscope analysis shows viral RNA in SARS-Cov-2 infected hamster OB glomeruli at 4dpi.

(**I** and **J**) Confocal image shows co-staining of endothelial cell marker CD31 and ACE2 in mouse (**I**) or hamster (**J**, ACE2 only) olfactory bulb.

(**K** and **L**) Immunostaining of CD45 and microglia marker IBA1 in the olfactory bulb of Control (**K**) or hACE2 mice (**L**) at 6 dpi. Arrowheads in **L** indicate IBA1 negative immune cells. In the hACE2 mouse strain, human ACE2 overexpression was driven by mouse Krt18 promoter. Scale bars: 100 µm (**A** and **D**), 50 µm (**I-L**), 20 µm (**H**).

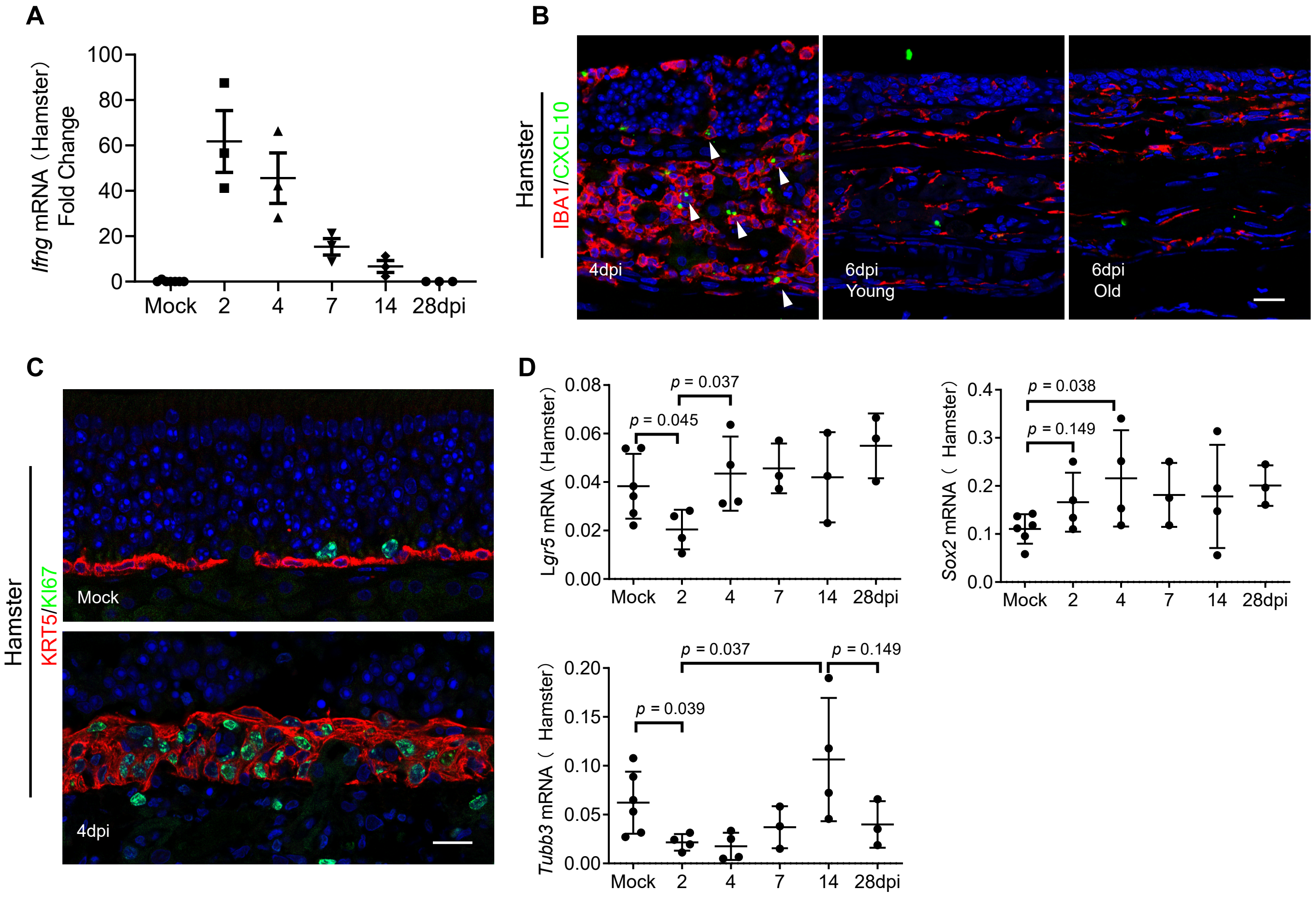


**Supplemental Figure 7. Regeneration of the olfactory epithelium**

(**A**) qPCR analysis of *Ifng* expression in turbinate tissues. SARS-CoV-2 infected hamsters were examined at indicated time points.

(**B**) Dynamic of IBA1+ macrophages infiltration and CXCL10 expression in hamster olfactory epithelium.

(**C**) Representative images show KRT5+ basal cells expressing proliferation marker KI67 on 4dpi in hamster olfactory epithelium.

(**D**) qPCR analysis of *Sox2*, *Lgr5*, and *Tubb3* expression in turbinate samples at indicated time points.

Data are represented as mean ± S.D. Statistical significance was determined by unpaired two-tailed t-test. Each data point represents an individual hamster (n = 3-6). Scale bars: 20 μm.

Supplemental Table 1. Characters of human olfactory epithelium biopsies

| No | Gender | Age | Condition | Biopsy Location | Figure | OE/RE | Infection or Nrp1 staining |
|---|---|---|---|---|---|---|---|
| 072220 | F | 33 | CRS | ST | Fig 1a, 4i | OE+RE | Infection |
| 072820 | M | 25 | CRS | ST | Fig 1e, 4i | OE+RE | Infection |
| 081220 | F | 55 | CRS | ST | Fig 1e, 4i | OE+RE | Infection |
| 090920 | M | 68 | CRS | ST | Fig 1e, 4i | OE+RE | Infection |
| 101920 | F | 72 | CRS | ST | Fig 1b, 4i | OE+RE | Infection |
| 091620 | F | 30 | CRS | ST | Fig 1e, 4i, 4j | OE+RE | Infection |
| 092120 | M | 54 | Control | ST | Fig 1e, 4i | OE+RE | Infection |
| 092120 | M | 54 | Control | Olfactory cleft | Fig 1c, 4i | OE | Infection |
| 091420 | F | 36 | Control | ST | Supp. Fig 1e | OE+RE | Mock |
| 100520 | M | 41 | Control | ST | NS | OE+RE | Mock |
| 081020 | M | 50 | Control | ST | NS | OE+RE | Mock |
| 061820 | F | 59 | CRS | ST | Fig 1e | RE | Infection |
| 062620 | F | 73 | Control | ST | Fig 1e | RE | Infection |
| 070820 | M | 67 | CRS | ST | Fig 1e | RE | Infection |
| 071620 | M | 48 | CRS | ST | Fig 1e | RE | Infection |
| 071720 | F | 71 | CRS | ST | Fig 1e | RE | Infection |
| 072120 | F | 58 | Control | ST | Fig 1e | RE | Infection |
| 072220 | M | 57 | CRS | ST | Fig 1e | RE | Infection |
| 073120 | F | 36 | Control | ST | Fig 1e | RE | Infection |
| 080520 | M | 58 | CRS | ST | Fig 1e | RE | Infection |
| 082620a | F | 57 | CRS | ST | Fig 1d,e | RE | Infection |
| 082620b | F | 62 | CRS | ST | Fig 1e | RE | Infection |
| 090220a | F | 56 | Control | ST+Septum | Fig 1e | RE | Infection |
| 090220b | M | 26 | Control | ST | Fig 1e | RE | Infection |
| 091420 | M | 72 | CRS | ST Left+Right | Fig 1e | RE | Infection |
| 091520a | F | 61 | CRS | ST | Fig 1e | RE | Infection |
| 091520b | M | 46 | CRS | ST | Fig 1e | RE | Infection |
| 091620 | M | 34 | CRS | ST | Fig 1e | RE | Infection |
| 093020 | F | 65 | CRS | ST | Fig 1e | RE | Infection |
| 102620 | M | 48 | CRS | ST | Fig 1e | RE | Infection |
| 101220 | F | 68 | Control | ST | Fig 1e | RE | Infection |
| 110220 | M | 63 | CRS | ST | Fig 1e | RE | Infection |
| 110420 | M | 77 | CRS | ST | Fig 1e | RE | Infection |
| 111020 | F | 41 | CRS | ST | Fig 1e | RE | Infection |
| 111320 | M | 65 | CRS | ST+Sinus | Fig 1e | RE | Infection |
| 040721 | F | 27 | Control | ST | Fig 4l | OE+RE | Nrp1 |
| 021721 | F | 30 | CRS | ST | Fig 4l,k | OE+RE | Nrp1 |
| 050721 | M | 20 | CRS | ST | Fig 4l | OE+RE | Nrp1 |
| 022221a | M | 68 | CRS | ST | Fig 4l | OE+RE | Nrp1 |
| 022221b | F | 69 | Control | ST | Fig 4l | OE+RE | Nrp1 |
| 090820 | F | 72 | CRS | ST | Fig 4l,k | OE+RE | Nrp1 |
| 040521 | M | 79 | Control | ST | Fig 4l | OE+RE | Nrp1 |

OE: olfactory epithelium; RE, respiratory epithelium; ST, superior turbinate; NS, image not shown.

**Supplemental Methods** (Chen et al, J Clin Invest. 2024):

The following primary antibodies was used: Rabbit anti-SARS-CoV-2 Nucleoprotein (NP, 1:200, Novus, NB100-56576), Rabbit anti- SARS-CoV-2 Nucleoprotein (1:500, GeneTex, GTX135357), Rabbit anti- SARS-CoV Nucleoprotein (1:1000, Rockland, 200-401-A50), Rabbit anti- SARS-CoV-2 Spike S (1:200, Sino Biological, 40150-R007), Goat anti-ACE2 (1:100, R&D, AF933, for human samples), Rabbit anti-ACE2 (1:100, Thermo, MA5-32307, for hamster samples), Goat anti-Neuropilin-1 (NRP1, 1:200, R&D, AF566) Mouse anti-Keratin 18 (KRT18, 1:500, Novus, NB500-306), Goat anti OMP (1:500, Wako, 544-10001), Mouse anti- alpha -Smooth Muscle Actin (a-SMA, 1:100, R&D MAB1420); Chicken anti-Vimentin (1:200, Novus NB300-223); Goat anti- forkhead box J1 (FOXJ1, 1:200, R&D AF3619); Mouse anti-FOX3/NEUN (1:1000, BioLegend, 834502); Rat anti-CD45 (1:300, Ebioscience, 14-0451-81), Rat anti-CD31(1:50, BD, 550274), Rabbit anti- Keratin 5 (KRT5, 1:500, Covance, PRB-160P), Chicken anti- KRT5 (1:500, BioLegend, 905904), Mouse anti pan-Keratin (1:200, BioLegend 628601), Mouse anti Tubulin beta 3 class III (TUBB3, 1:300, BioLegend, 801203, clone Tuj1), Rabbit anti Ionized calcium binding adapter molecule 1 (IBA1, 1:500, Wako, 019-19741), Rabbit anti-Cleaved Caspase-3 (CASP3, 1:300, Cell signaling, 9664), Rat anti DC-associated C-type lectin-2 (DECTIN-2, 1:200, Bio-Rad, MCA2415T), and Goat anti-CXCL10 (1:100, R&D, AF-466-NA).

After washing in PBS three times, the tissue sections were incubated with Alexa Fluor conjugated, highly cross-adsorbed, secondary antibodies along with DAPI for nuclear counterstaining. The donkey-derived Alexa Fluor-conjugated secondary antibodies included anti-mouse 488 (A21202, Invitrogen); anti-Rat 488 (A21208, Invitrogen); anti-Rabbit 488 (A21206, Invitrogen); anti-Rabbit 546 (A10040, Invitrogen); anti-Goat 488 (A32814, Invitrogen); anti-Goat 546 (A11056, Invitrogen); anti-Chicken 488 (SAB4600031, Sigma).

**Supplementary Table 2.** SYBR Green PCR primer sequences of hamster or mouse genes

Hamster

| Gene | Forward | Reverse |
|---|---|---|
| *Ace2* | TGGTGGGAGATGAAGCGAGA | GAACAGAGCTGCAGGGTCAC |
| *Omp* | CAGAAGCTGCAGTTCGACCG | CAGAAGATTGCGGCAGGGTC |
| *Ifng* | TAATGCACACCACACGTTGC | AAGACGAGGTCCCCTCCATT |
| *Gapdh* | GTGGAGCCAAGAGGGTCATC | GGTTCACACCCATCACAAACAT |

Mouse

| Gene | Forward | Reverse |
|---|---|---|
| *Nrp1* | CAGTGGCACAGGTGATGACT | ACCGTATGTCGGGAACTCTGAT |
| *Ace2* | CCATTGGTCTTCTGCCATCCG | CCAACGATCTCCCGCTTCATC |
| *Gapdh* | TCAATGAAGGGGTCGTTGAT | CGTCCCGTAGACAAAATGGT |
